# The design of a Bayesian adaptive clinical trial of tranexamic acid in severely injured children

**DOI:** 10.1186/s13063-021-05737-0

**Published:** 2021-11-04

**Authors:** John M. VanBuren, T. Charles Casper, Daniel K. Nishijima, Nathan Kuppermann, Roger J. Lewis, J. Michael Dean, Anna McGlothlin, For the TIC-TOC Collaborators of the Pediatric Emergency Care Applied Research Network (PECARN)

**Affiliations:** 1 Department of Pediatrics, University of Utah School of Medicine, 295 Chipeta Way, Salt Lake City, UT 84108 USA; 2 Department of Emergency Medicine, University of California, Davis School of Medicine, Sacramento, CA 95817 USA; 3 Department of Pediatrics, University of California, Davis School of Medicine, Sacramento, CA 95817 USA; 4 Department of Emergency Medicine, Harbor-UCLA Medical Center, Torrance, CA 90509 USA; 5 Berry Consultants, LLC, Austin, TX 78746 USA

**Keywords:** Bayesian statistics, Tranexamic acid, Pediatrics, Adaptive clinical trial design, Response-adaptive randomization, Dose-response, Trauma

## Abstract

**Background:**

Trauma is the leading cause of death and disability in children in the USA. Tranexamic acid (TXA) reduces the blood transfusion requirements in adults and children during surgery. Several studies have evaluated TXA in adults with hemorrhagic trauma, but no randomized controlled trials have occurred in children with trauma. We propose a Bayesian adaptive clinical trial to investigate TXA in children with brain and/or torso hemorrhagic trauma.

**Methods/design:**

We designed a double-blind, Bayesian adaptive clinical trial that will enroll up to 2000 patients. We extend the traditional *E*_*max*_ dose-response model to incorporate a hierarchical structure so multiple doses of TXA can be evaluated in different injury populations (isolated head injury, isolated torso injury, or both head and torso injury). Up to 3 doses of TXA (15 mg/kg, 30 mg/kg, and 45 mg/kg bolus doses) will be compared to placebo. Equal allocation between placebo, 15 mg/kg, and 30 mg/kg will be used for an initial period within each injury group. Depending on the dose-response curve, the 45 mg/kg arm may open in an injury group if there is a trend towards increasing efficacy based on the observed relationship using the data from the lower doses. Response-adaptive randomization allows each injury group to differ in allocation proportions of TXA so an optimal dose can be identified for each injury group. Frequent interim stopping periods are included to evaluate efficacy and futility. The statistical design is evaluated through extensive simulations to determine the operating characteristics in several plausible scenarios. This trial achieves adequate power in each injury group.

**Discussion:**

This trial design evaluating TXA in pediatric hemorrhagic trauma allows for three separate injury populations to be analyzed and compared within a single study framework. Individual conclusions regarding optimal dosing of TXA can be made within each injury group. Identifying the optimal dose of TXA, if any, for various injury types in childhood may reduce death and disability.

**Supplementary Information:**

The online version contains supplementary material available at 10.1186/s13063-021-05737-0.

## Background

Trauma is the leading cause of death and disability in children in the USA [1]. In the initial 24 h after injury, hemorrhage is the leading cause of death [2]. Tranexamic acid (TXA) is an antifibrinolytic lysine analog that blocks the conversion of plasminogen to plasmin thereby helping to attenuate bleeding. TXA reduces the blood transfusion requirements in adults and children during surgery [3–7]. The effectiveness of TXA in the surgical setting led to the Clinical Randomization of an Antifibrinolytic in Significant Hemorrhage (CRASH-2) and CRASH-3 trials, two separate international randomized controlled trials of early administration of TXA to adults with hemorrhagic torso trauma (CRASH-2) and hemorrhagic brain injuries (CRASH-3) [8, 9]. In CRASH-2, TXA reduce mortality with no increase in adverse events compared to placebo. In CRASH-3, patients randomized to TXA had lower mortality, but it was not statistically significant. In addition, a post hoc analysis of the CRASH-2 trial demonstrated improved functional outcomes associated with TXA use [10].

Several challenges exist for studying the efficacy of TXA in children. First, children are different from adults with regard to anatomy, physiology, and metabolism, all of which may affect the efficacy and safety profiles of TXA differently in children than it does in adults, creating uncertainty about the optimal dosing in children. Second, the efficacy and safety of TXA may be different in children with torso/abdominal hemorrhage than in children with brain hemorrhage, and children with both torso and brain hemorrhage (polytrauma) may also respond differently. Because severe hemorrhagic injury is less frequent in children than in adults, conducting separate clinical trials for each hemorrhagic condition in children (isolated brain, isolated torso, and polytrauma injuries) would be inefficient and costly. However, conducting a trial that combines all severe pediatric hemorrhagic injury groups in a single analysis could neglect important systematic differences in treatment effect that may exist between the groups. A pilot study enrolling 31 patients was successfully completed demonstrating the feasibility of patient consent, enrollment, and follow-up procedures [11], but a larger study is needed to assess efficacy.

Bayesian adaptive clinical trials have been successfully used to help study rare diseases and infrequent conditions [12, 13]. Adaptive designs allow for the features of the study to change in a pre-specified way based on accumulating data during the trial. These approaches are becoming more common, and the FDA has recently released guidance on adaptive designs in industry clinical trials [14].

The Traumatic Injury Clinical Trial Evaluating Tranexamic Acid in Children (TIC-TOC) trial is a Bayesian adaptive prospective, double-blinded, placebo-controlled, multicenter adaptive trial to assess the efficacy and safety of TXA in children with severe hemorrhagic injury to the brain and/or torso. The trial includes several innovative features designed to improve the efficiency of the trial and address inherent challenges. This trial will test the efficacy of at least two doses of TXA in children with hemorrhagic brain and/or torso injuries. The use of dose-response modeling and response-adaptive randomization allow efficient investigation of several doses, including a staggered approach to initiating the highest dose, which has been successfully performed in other studies [15, 16]. Additionally, we will incorporate a hierarchical model that leverages data across the injury groups for efficient estimation and inference. Finally, we propose a novel patient-centered endpoint that captures both the outcome and its trajectory.

## Methods/study design

The Traumatic Injury Clinical Trial Evaluating Tranexamic Acid in Children (TIC-TOC) study will be a multicenter trial in children with hemorrhagic brain and/or torso injuries. Study drug (TXA or placebo) will be given within 3 h of injury starting with a 30-min bolus dose followed by an 8-h infusion.

The trial will enroll up to 2000 children whose hemorrhagic injuries are classified as “Brain,” “Torso,” or “Both” at 30–40 academic level 1 trauma centers in the USA. The sample size will be adaptively determined by frequent interim analyses that will be performed after 500, 750, 1000, 1250, 1500, and 1750 patients are randomized. Initially, patients will be randomized equally among three study arms: placebo, TXA 15 mg/kg bolus dose then a 2 mg/kg/h infusion, and TXA 30 mg/kg bolus dose then a 4 mg/kg/h infusion, with the possibility of initiating a TXA 45 mg/kg bolus dose then a 6 mg/kg/h infusion arm later in the trial based on accumulating data. At each interim analysis, the randomization probabilities will be adjusted to preferentially allocate patients to better performing dosing arms, while the placebo arm probability will stay fixed. The trial will use a Bayesian hierarchical model that allows the estimates of the treatment effect for each of the injury types to be informed by the data accumulated from all injury types. SPIRIT reporting guidelines were used in this manuscript [17].

### Primary outcome measure: the Pediatric Quality of Life Inventory (PedsQL) area under the curve

The Pediatric Quality of Life Inventory (PedsQL) is a survey that assesses various quality aspects of a child’s life [18]. There are different versions based on a child’s age to account for development stages. The total score for all age versions ranges from 0 to 100 where higher scores correspond to a better quality of life, and the instrument has been validated across age groups. In the TIC-TOC study, the PedsQL survey will be administered 1 week, 1 month, 3 months, and 6 months after injury. The primary outcome in the TIC-TOC trial will be the area under the curve (AUC) of these surveys which results in a continuous outcome measure ranging between 0 and 100 [19]. AUC is commonly used for the analysis of longitudinal quality of life surveys and our approach to the derivation of AUC is similar to published literature [20, 21]. All four PedsQL measures (at the various time points) will be used in the calculation of the AUC for the Brain and the Both injury groups. Most patients with Torso injuries are expected to recover by one month, regardless of what treatment the patient receives. For this reason, in the Torso injury group, only the 1 week and 1 month surveys will be included in the primary outcome calculation. The AUC captures both the amount of recovery as well as the rate of recovery within a single measurement. It also allows a varying number of time points across injury groups to be incorporated into a single measure that is comparable across injury groups. The AUC may be calculated in the presence of missing follow-up visit surveys if the PedsQL is observed for at least one timepoint using the trapezoid rule. If only one follow-up PedsQL outcome is observed, that value is assigned to the AUC. For any single timepoint, a difference between PedsQL surveys of 4.5 units has been considered to represent a clinically meaningful difference [22]. We have applied this 4.5 clinically meaningful difference to the AUC outcome as well.

### Statistical model

A dose-response model is used to characterize the relationship between the dose of TXA and outcomes as defined by the PedsQL AUC. The selected model is a hyperbolic *E*_*max*_ model [23], which is commonly used in dose-ranging studies in all stages of drug development [24]. The model has the distinguishing feature that it is monotonic; thus, higher doses are expected to have greater difference from placebo in average PedsQL AUC values.

The hyperbolic *E*_*max*_ model is typically used within a single population of interest. Since the TIC-TOC trial investigates three distinct, yet related, populations (Brain injury, Torso injury, and Both), we extend the model to accommodate multiple injury types. We introduce a hierarchical structure [25] to the model that allows inferences to be drawn within each injury group while leveraging data from all patients in the trial. This integration (“borrowing”) of information across injury groups is a key feature of the design and is especially critical for the Both injury group because the sample size is expected to be substantially smaller than for the other injury groups. The hierarchical model is specified a priori but the behavior of the model is dynamic in that it will borrow or share more information across injury groups when the observed effects of TXA compared to placebo are similar across injury groups, while the model results in less borrowing and more independent estimates when there is a more heterogeneous result observed. In addition, this borrowing reduces the overall probability of making at least one false claim of success under the scenario in which none of the injury groups has true benefit (the global null). When there is no effect of TXA in any injury group, the borrowing mitigates the impact of spurious results within a particular arm of an injury group. A benefit of the hierarchical model is a greater precision of estimates and better decisions regarding benefit of TXA in each injury type [25]. Details of the statistical model, including prior distributions, are provided in the supplement (Adaptive Design Report).

All inferences about the primary outcome will be based on this prespecified model, which will be periodically updated as described below using a Bayesian framework as data accrues during the trial. Probabilities derived from this model will guide all adaptive decisions within each injury type, including the response-adaptive randomization, the evaluation of pre-specified stopping rules, and whether to open a higher dose arm. The adaptive design rules are described below.

### Response adaptive randomization

Response-adaptive randomization allows the proportion of patients allocated to the different dosage arms to change throughout the trial based on accumulating data. There are different methods and algorithms used to implement response-adaptive randomization; our method is similar to that described in Trippa et al. [26]. From the statistical model, we determine which dose is estimated to have at least 80% of the treatment effect (*ED*_80_) attributable to the maximum dose (i.e., 45 mg/kg). The randomization probabilities within each injury type will be proportional to the probability that each dose is the *ED*_80_ within that injury type. By targeting the *ED*_80_, the algorithm will tend to back away from the highest dose whenever a lower dose is available that maintains most of the treatment effect of the higher dose. This strategy helps minimize the number of children exposed unnecessarily to high doses.

Response-adaptive randomization is implemented starting after 500 total patients have been enrolled. After that, the proportions allocated to each arm within each injury are updated every time an additional 250 patients have been enrolled (i.e., after 500, 750, 1000, 1250, 1500, and 1750 patients). The allocation proportions across arms can differ between the injury groups. Only individuals who have an observed outcome will contribute to the response adaptive randomization. This means that recently randomized patients without an observed outcome will not contribute when the study reaches the interim sample size (e.g., 500), but will contribute their information to the following interim analysis assuming they have an observed outcome at that point. Because this study is conducted in a blinded fashion and study drug is given over 8 h, patients who were randomized to an arm that is suspended will have completed treatment by the time the allocations are updated and will not be notified of the suspension.

An additional constraint is applied to prevent the algorithm from assigning patients to underperforming doses. Within any injury group, if the randomization allocation probability drops below 10% for a given dose, that dose is temporarily dropped from the randomization sequence for that injury group. At future interim looks, it is possible that a dropped dose could re-enter the allocation proportions based on the additional accumulating data. The placebo arm is forced to remain in the study and is not allowed to be dropped. If the response-adaptive randomization results in only two arms being used at a single time within an injury group, the allocations are forced to be 50% placebo and 50% to the TXA dose being used. Otherwise, the placebo arm allocation probability is fixed at 33% throughout the trial.

### Adding the high dose

A common concern in drug development is that too narrow of a dose range is explored, which limits the selection of doses to continue on to the later phase trials [27]. If too low of a dose is selected, this mistake increases the risk of failure as the full potential of the drug is not considered. The exploration of higher doses needs to be appropriately balanced with any safety concerns. Although TXA has been demonstrated to be safe in other populations, the TIC-TOC trial will start enrolling in each injury group with three arms (placebo [0 mg/kg], TXA 15 mg/kg, and 30 mg/kg). Within a single injury group, we will add a 45 mg/kg arm of TXA if both of the following conditions are met for that injury group:

- There is at least a 50% posterior probability that the model-estimated difference in AUC between the 45 mg/kg arm and placebo is greater than or equal to 4.5 quality of life units for that injury group (the minimum clinically meaningful difference) based on extrapolating the results seen in the 15 mg/kg and 30 mg/kg arms, and
- There are no safety concerns for a higher dose identified by the Data and Safety Monitoring Board based on current data.

If the 45 mg/kg dosing arm is opened within an injury group, the initial randomization probability for that dose will be capped at no more than 20% until the probabilities are re-evaluated at the next interim look. This cap is instituted to ensure that preliminary safety data can be collected on the 45 mg/kg arm within an injury group before a larger proportion of patients is randomized to that dose. After the 45 mg/kg dosing arm has been initiated for at least one interim period, the maximum allocation proportion for the 45 mg/kg arm increases to 50%.

### Stopping rules and decision boundaries

The final analysis for each injury type does not occur until after all enrolled patients in that injury type have reached the last PedsQL survey timepoint. At the final analysis, an injury group will be meeting the criteria for efficacy if there is sufficiently high probability that the dose-response model has a positive slope. We will use the probability that each dose has at least 80% of the treatment effect (*ED*_80_) attributable to the maximum dose when updating the allocation sequence. However, for the final analysis, our goal is to evaluate whether there is an efficacy signal of TXA as a whole which is why we evaluate the slope of the curve.

An injury group may stop enrollment early either for reaching a sample size cap (1600, 900, and 300 patients, respectively for the Brain, Torso, and Both groups), for futility, or for the expectation of success. Stopping for efficacy and futility will be considered after 1000 patients are enrolled and after each additional 250 patients thereafter (i.e., after 1000, 1250, 1500, 1750, and 2000 [maximum sample size] total patients are enrolled). When an injury group stops for efficacy or futility, the official analysis will be performed once all patients randomized in that injury group have reached their last follow-up period. At that point, the data will be locked and the final analysis for the injury group will be performed with all new accumulated data (even from other injury groups).

From the model, we compute the posterior probability of a positive treatment effect within each injury group. This probability is evaluated against the boundaries in Table 1. For example, after 1000 patients have been randomized, the Torso group may stop accrual for expected success if the probability of a positive treatment effect at the current sample size is at least 0.980 or stop for futility if the probability of a positive treatment effect at the current sample size is lower than 0.05.

**Table 1:** Boundaries of posterior probabilities of efficacy for stopping accrual and for final analysis decisions

Total sample size	Brain		Torso		Both	
	Efficacy	Futility	Efficacy	Futility	Efficacy	Futility
1000	0.990	0.050	0.980	0.050	0.975	0.050
1250	0.990	0.100	0.980	0.100	0.975	0.100
1500	0.990	0.150	0.980	0.150	0.975	0.150
1750	0.990	0.200	0.980	0.200	0.975	0.200
Final analysis	0.981	0.250	0.978	0.250	0.975	0.250

The stopping boundaries were selected a priori to control for the probability of a false positive conclusion within an injury type below 2.5% (one-sided) under the global null scenario (no effect in the Brain, Torso, nor Both injury groups), which was verified through simulation. When a stopping boundary is crossed, recruitment is stopped for that injury group, and the patients currently enrolled in that injury group continue to have follow-up until all individuals in that group have been evaluated at all follow-up time periods. Due to the infrequency of patients in the Both injuries group, if both the Brain and Torso injury groups stop enrollment for any reason, we will stop recruitment in the Both injuries group regardless of the current effect size. This is to prevent the trial from continuing for an infeasible amount of time. For similar reasons, if either the Brain injury group or the Torso injury group meet stopping boundaries, the remaining enrolling injury groups are limited to a maximum sample size cap (to not excessively prolong the study). Based on clinical knowledge about the proportions that these injuries occur (assumed to be 60% Brain injuries, 30% Torso injuries, 10% Both injuries), the maximum sample size for the Brain, Torso, and Both groups are set to 1600, 900, and 300 patients, respectively. These caps allow for fluctuation in the assumed injury distribution since the maximum total sample size stays fixed at 2000 patients.

### Final analysis of the both group

Due to the lower prevalence of children with both head and torso injuries, we do not expect that the trial will have sufficient power to detect benefit in this group on its own at interim analyses at the assumed effect sizes of TXA, even with the hierarchical model. At the end of the trial, if both the Brain and Torso groups are shown to be efficacious and the Both group has not yet crossed a stopping boundary, a separate hyperbolic *E*_*max*_ model will be performed on the Both injury group. For this model, informative priors will be constructed using the estimates obtained in the Brain and Torso groups. Additional details are provided in the supplement (Adaptive Design Report).

### Simulations

**Fig. 1:**
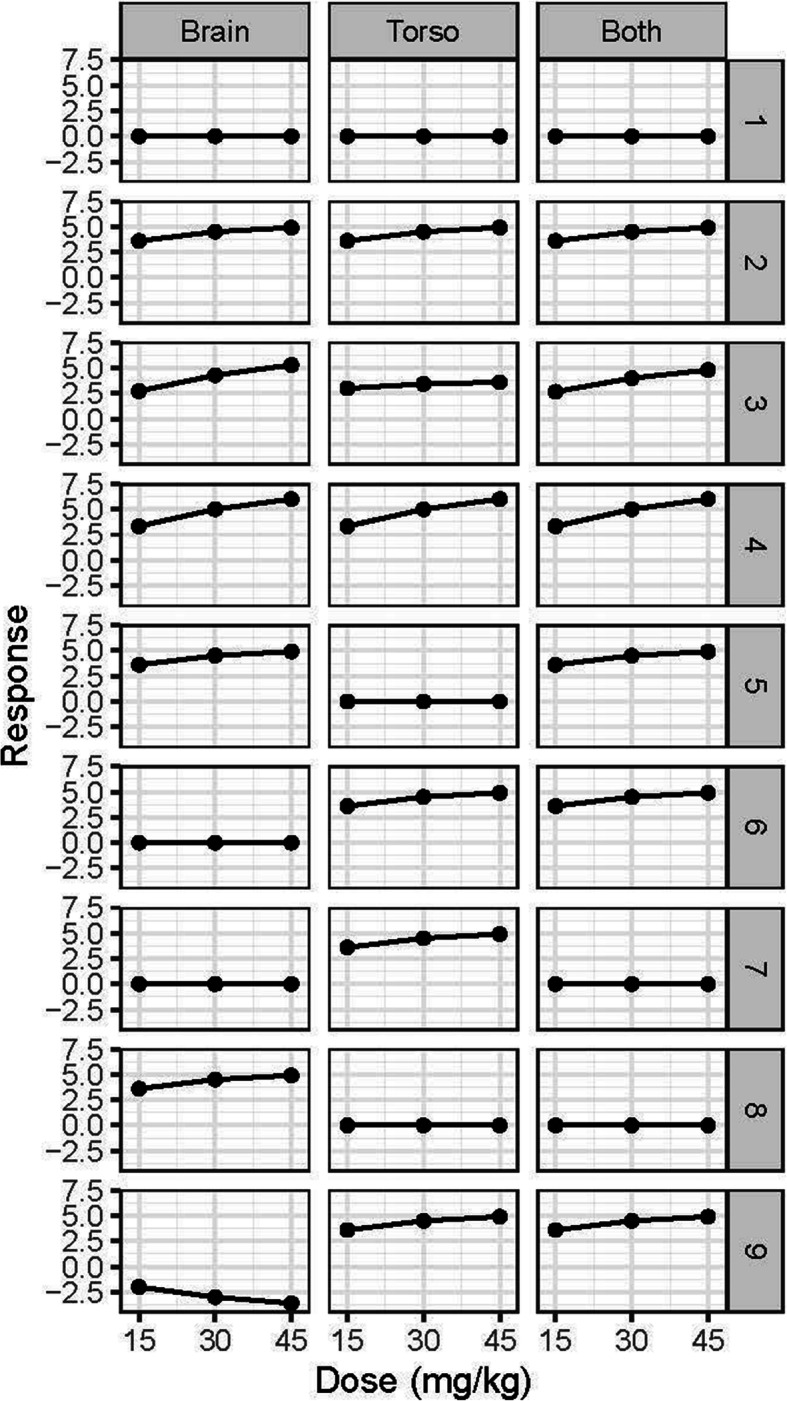
Nine scenarios are presented to understand the operating characteristics of the model. Effect sizes for the PedsQL AUC compared to the placebo arm are plotted for each dose for the Brain, Torso, and Both injury groups. Lines above zero on the vertical axis indicate efficacious scenarios where flat lines at zero indicate the null scenario (no benefit). Lines below zero on the vertical axis indicate harmful scenarios

To evaluate the behavior of the adaptive design, we performed computer simulations of the trial across a range of plausible scenarios. We created a set of hypothetical dose-response relationships for PedsQL AUC for each injury type. For each of the scenarios displayed in Fig. 1, we simulated 5000 trials. In each trial, we generated PedsQL AUC scores according to the truth for the scenario and had the computer conduct the design as specified above. This process was repeated, and the behavior of each “virtual trial” was tracked, including the number of patients assigned to each dose within each injury type, when accrual was stopped and the reason, and the selected dose, if any, for each injury type. These operating characteristics were then summarized across all simulated trials for each scenario.

The effect sizes for the Brain, Torso, and Both groups are shown in their respective columns. The lines plotted display the treatment effect (the difference between each dose and placebo). Flat lines at 0 indicate the null effect (i.e., no difference between TXA and placebo) and is represented in scenario 1. Scenarios 2 through 4 indicate varying levels of efficacy of TXA. Scenarios 5 through 8 represent mixed situations and are used to understand the impact of the hierarchical model when TXA is efficacious in at least one injury group but not in at least one other. Scenario 9 represents a situation where TXA is harmful in one injury type and efficacious in the other two.

### Operating characteristics

Using these stopping boundaries, the following operating characteristics were determined through simulation among nine scenarios. For our simulations, power was defined within an injury group as the probability of successfully identifying TXA is superior to placebo when that is the case based on the estimated dose response curve. Due to the borrowing of information across injury groups, the thresholds are more liberal (lower thresholds) compared to traditional boundaries. This is because it is more difficult to reject the null hypothesis with the borrowing unless you are seeing a large signal in one injury group or a consistently positive signal across all three injury groups (i.e., the borrowing smooths out spurious results within a particular arm of an injury group). The one-sided type I error rate is preserved in the Brain (2.1%), Torso (2.2%), and Both (1.4%) injury groups under the null scenario.

Under the hypothesized effect size (scenario 2), there is 94.9% power in the Brain group, 82.9% power in the Torso group, and 64.4% power in the Both group to detect a difference in PedsQL AUCs between placebo and TXA. The operating characteristics of the other scenarios can be seen in Table 2.

**Table 2:** Operating characteristics of nine different scenarios

Scenario	Power			Probability of stopping the entire trial at specific interim looks					Probability of opening 45 mg/kg dose		
	Brain^†^	Torso^†^	Both^†^	1000	1250	1500	1750	2000	Brain	Torso	Both
1	*2.1%*	*2.2%*	*1.4%*	0.3%	1.8%	2.5%	4.4%	91.0%	21.5%	20.6%	21.7%
2	**94.9%**	**82.9%**	**64.4%**	16.9%	22.5%	19.8%	15.9%	24.8%	73.9%	70.7%	63.9%
3	92.3%	64.6%	52.4%	10.1%	15.2%	15.9%	16.7%	42.1%	72.5%	65.0%	65.1%
4	97.0%	89.0%	73.1%	23.8%	27.8%	20.3%	14.3%	13.7%	79.3%	76.1%	68.7%
5	92.5%	*5.0%*	39.0%	2.1%	4.2%	5.6%	18.2%	69.9%	67.3%	35.5%	59.8%
6	*4.4%*	65.9%	*30.5%*	0.9%	2.4%	4.6%	5.4%	86.6%	30.4%	63.4%	56.4%
7	*3.2%*	61.0%	*3.2%*	0.9%	2.2%	4.2%	5.9%	86.9%	27.0%	57.7%	30.6%
8	90.6%	*4.3%*	*5.3%*	1.9%	4.0%	5.6%	11.4%	77.2%	64.7%	31.6%	34.7%
9	0.0%	71.4%	24.7%	3.5%	11.6%	18.6%	20.1%	46.1%	9.4%	53.0%	41.2%

^†^Italicized cells indicate the one-sided type I error rate in those scenarios. Bolded cells indicate the postulated scenario

The probability of stopping the entire trial at each interim look assessing futility and efficacy are also displayed (meaning all three injury groups have stopped enrollment). Under the null scenario, there is a 91% chance that the trial will continue to enroll 2000 patients and approximately a 20% chance in each injury group that the 45 mg/kg arm will be opened. Under the hypothesized effect (scenario 2), the probability of stopping at each interim look ranges between approximately 15% and approximately 25%. There is a 64% to 74% probability in each injury group that the 45 mg/kg dosing arm will be opened. In scenario 3, there are varying effects of TXA across the injury groups. As seen in Fig. 1, in this scenario, TXA is less efficacious in the Torso group compared to the Brain and Both groups. This corresponds to a substantial decrease in power for the Torso group and only a minor decrease in power in the Brain group. The inability to claim benefit in the Torso group also results in a decrease in power for the Both group (due to the hierarchical model). In the settings where TXA is more efficacious than hypothesized (scenario 4), the power greatly increases for identifying benefit in the Torso and Both injury groups and there is a larger likelihood that the trial will stop early. Due to the borrowing of information across the injury groups, the one-sided type I errors are mildly inflated in scenarios when there is an effect in at least one group and no effect in at least one other group (scenarios 5 through 8). In the scenario where there is a harmful effect in the Brain group but efficacious in Torso and Both, we have less than a 0.1% chance that we conclude TXA is efficacious in the Brain injury group (scenario 9). The Torso group maintains adequate power (71.4%) to detect an effect of TXA. This scenario demonstrates how borrowing is reduced when the observed effects are conflicting.

### Expected sample sizes by injury group and dose

**Fig. 2:**
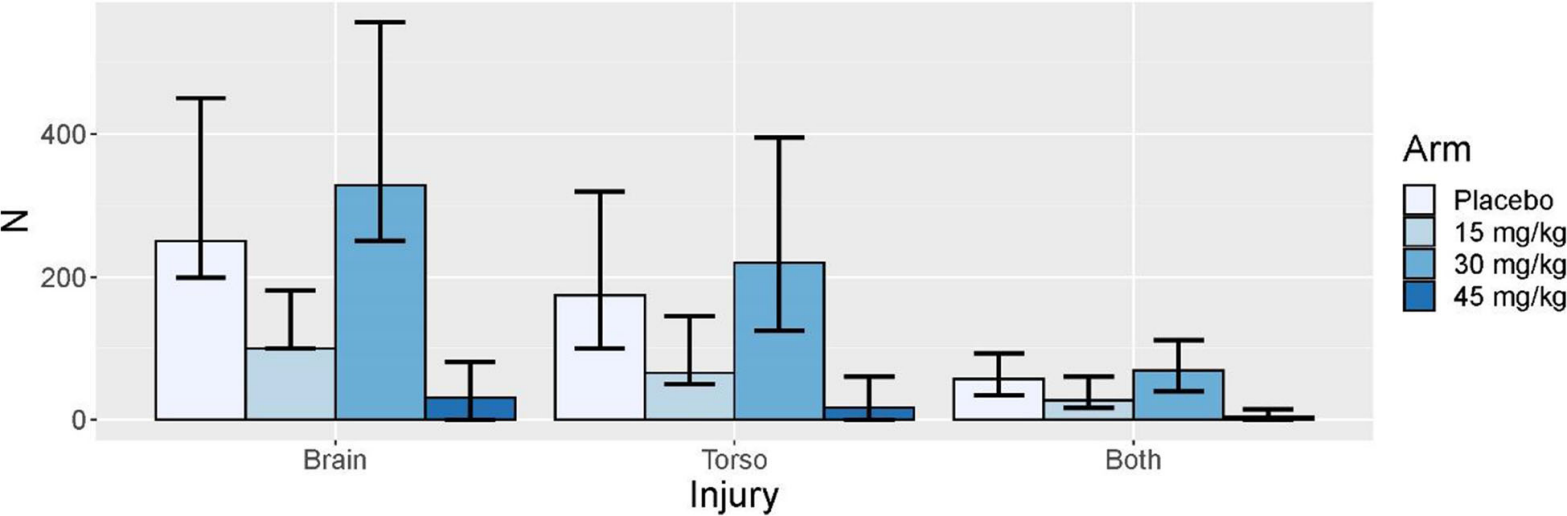
Summaries of expected sample sizes by injury group and arm for the hypothesized scenario (scenario 2 [dose responsive benefit seen in all three injury groups]). The bars display the median and the lines display the 10th and 90th percentile of the expected sample sizes across simulations

Due to the response-adaptive randomization, the proportions of patients in each study dosing arm change based on the accumulating data. For each simulated trial, we record the sample size per dose at the time that an injury group stops accrual. The median sample size for each study arm within each injury group is displayed for the hypothesized scenario (scenario 2) in Fig. 2. The bars extending from each median display the 10th and 90th percentile sample sizes among the simulations. Because response adaptive randomization does not begin until 500 patients have been randomized, there is a minimum number of patients that are expected to be observed for the placebo, 15 mg/kg, and 30 mg/kg dosing arms in the three injury groups, regardless of the effect size. In the hypothesized scenario, the assumed effect sizes are 3.6 units (15 mg/kg), 4.5 units (30 mg/kg), and 4.9 units (45 mg/kg), so that the ED80 would be the minimum dose that results in an effect size of at least 3.9 quality of life units in AUC (i.e., 4.9 effect size at 45 mg/kg * 80% effectiveness = 3.9 quality of life threshold). In this case, the 30 mg/kg dose would be the correct ED80 in each injury group. As expected and demonstrated in Fig. 2, the response-adaptive randomization favors the 30 mg/kg arm.

**Fig. 3:**
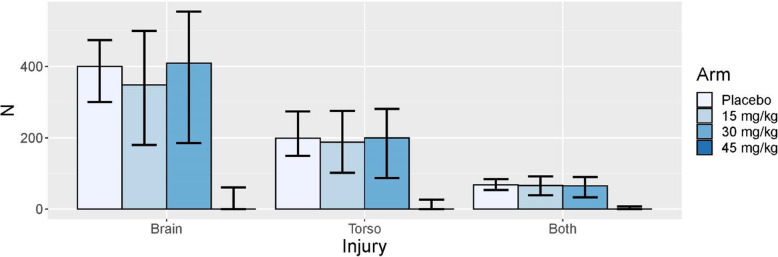
Summaries of expected sample sizes by injury group and arm for the null scenario (scenario 1 [no benefit or harm seen with TXA across injury groups]). The bars display the median and the lines display the 10th and 90th percentile of the expected sample sizes

Figure 3 displays the average sample sizes under the null scenario (scenario 1). The adaptive randomization tends to apply roughly equal allocation across the dosing arms. Even though the 45 mg/kg dosing arm is opened in approximately 20% of the null simulations as seen in Table 2, very few patients are actually allocated to the 45 mg/kg dosing arm. This is partly due to the restrictions that are placed on the allocation probabilities to the high dose once it is introduced.

## Discussion

The TIC-TOC trial will provide an important addition to medical research for the treatment of children with hemorrhagic injuries. Although there are substantial data regarding the effectiveness of TXA for mitigating bleeding in both adults and children during surgery [3–7], and a mortality benefit of TXA for adults with hemorrhagic torso [8] and brain injuries [9], there have been no clinical trials of TXA for injured children. This critical gap in knowledge has substantial implications. If TXA were found to be efficacious in children with hemorrhagic injuries, it would likely be a safe, inexpensive adjunct in the management of injured children worldwide. If not found to be efficacious, or if safety issues outweighed its benefits, TXA should not be used for routine care of children with hemorrhagic injuries.

This trial design offers solutions to the unique challenges of investigating TXA in injured children by making the most efficient use of valuable patient resources to answer several research questions. These include whether TXA provides benefit in three different injury groups, and which dose should be used in this setting. The incorporation of early stopping rules allows enrollment to end within an injury group once the research question has been answered or is futile.

A traditional clinical trial might either limit enrollment to a single injury type, or would pool data together across injury types, assuming a common treatment effect. Our Bayesian adaptive design incorporates a statistical model that will provide a distinct treatment effect estimate for each injury type that leverages data for all injury types, to the extent that the treatment effects are similar. Our hierarchical model considers the three injury types to be unordered groups. A more sophisticated model might be conceived that better reflects the nature of the groups, that is, that the Both group overlaps the Brain and Torso groups. Designing the study with the Bayesian framework allows for easy interpretation of the study results compared to what is typically provided by frequentist analyses [28].

The response-adaptive randomization provides a more ethical approach because patients within the trial are more likely to be assigned to the study arm that appears to be more efficacious at the time. There have been simulation studies that suggest that response-adaptive randomization has limitations and unintentional consequences to the study design. However, this has been predominantly in two-arm trial settings in which the control arm allocation is allowed to decrease or when appropriate tuning parameters are not chosen [29–31]. Trippa et al. [26] demonstrate that response-adaptive randomization can require fewer overall patients compared to a fixed design while maintaining a certain level of statistical power. A detailed discussion and simulation study showing the benefit of response-adaptive randomization in scenarios like what is used in the TIC-TOC study design can be found elsewhere [32]. The investigators of that study compared various versions of response-adaptive randomization previously published and discuss the effect response-adaptive randomization has on power, sample size, and arm selection. Another limitation of response-adaptive randomization is the presence of time trends. In particular, if there is a time effect for the treatment of patients, then decisions made earlier in a trial may not reflect what should have occurred at a later time (which might result in parameter drift in the ongoing model estimation). For this study population, the management of pediatric hemorrhagic trauma patients has not changed significantly in years. Due to the severity of the patient’s situation, they are prioritized upon emergency department arrival so we do not believe there will be concerns for time trends during enrollment. In the unexpected situation where a time trend is observed, a time variable will be incorporated into the regression as described in Robertson [33].

In pediatric surgery, up to 100 mg/kg doses of TXA are used [6]. Therefore, there is clinical precedent to use higher doses than 30 mg/kg of TXA in children. However, we are taking a cautious approach, starting with lower doses and adaptively expanding randomization to the higher dose. Additionally, by targeting the dose that is estimated to have at least 80% of the treatment effect attributable to the maximum dose, the response-adaptive randomization algorithm will favor lower doses whenever they provide comparable efficacy to higher doses. There are potential limitations when adding a new dose to a study as illustrated by Lee [34] such as time trends, type I error inflation, and implementation challenges for existing patients. As discussed above, the presence of time trends is not expected in this population. This statistical design evaluates the dose response curve compared to placebo instead of a separate comparison for each individual dose which removes a common multiplicity concern. In addition, the type I error rates in each injury group have been verified through simulation. The study is implemented in a blinded fashion so concerns about the addition of the 45 mg/kg arm is mitigated by the fact that only certain unblinded personnel will be aware of what each patient has received. We have chosen to restrict the maximum allowed proportion initially allocated to the 45 mg/kg arm to 20%, recognizing that this does not allow a substantial number of patients to be monitored at the 45 mg/kg arm for safety events. In the rare event that there is a safety concern with 45 mg/kg, we would likely observe a safety signal among the placebo, 15 mg/kg, and 30 mg/kg arms earlier in the trial before that arm is opened during a regularly scheduled data and safety monitoring board review.

In multi-arm trials, using a dose-response curve has many benefits compared to estimating the response for each dose separately [35]. In a traditional trial, patients may be allocated to a higher dose immediately which could lead to safety concerns or which might not consider a higher dose and lose the possibility of detecting the treatment effect of an optimal dose. Using the Bayesian adaptive approach, we open a higher dose in a systematic fashion based on accumulating data. This is conceptually similar to phase I dose escalation trials [36]. The model also adjusts for multiplicities so there is no penalty for multiple arms. Incorporating this structure reduces the variances when looking at trends between doses.

The hyperbolic *E*_*max*_ model has been extended in previous settings, for example to accommodate non-monotonic dose-response relationships [37]. The TIC-TOC study will implement a different extension of the hyperbolic *E*_*max*_ model to allow for incorporation of multiple injury groups into a single analysis. The hierarchical structure allows for the more robust inference of less prevalent injuries such as the group of children with both brain and torso injuries. Other methods have implemented these structures in the past [12, 25].

During the process of designing this trial, we considered a range of parameters controlling the extent of borrowing across injury types through the hierarchical model. We also explored different stopping boundaries. Due to the safety profile of TXA and what has been established regarding the efficacy signal in adults, we designed this trial to provide a definitive answer (efficacy or futility) of whether TXA should be given to children who experience a traumatic hemorrhagic injury. We chose a less aggressive futility rule to provide the robust evidence needed to avoid using TXA if it does not improve outcomes for children in emergency situations. After considering the operating characteristics of each of these design variants, the design described above was selected, as it provided adequate power for the anticipated treatment effect scenario while not inflating the false positive rate beyond a reasonable degree due to the borrowing across injury types. These selected stopping boundaries provide operating characteristics with reasonable trade-offs in the sample size distributions.

At the end of the trial, if efficacy has been declared in the Torso injury and Brain injury groups, but not yet in the Both injury group, we provide a method for analyzing the Both group to reach a more appropriate conclusion about the potential benefit of TXA in that arm. This process incorporates clinical reasoning into the statistical model. In a scenario in which efficacy is demonstrated in the Torso and the Brain injury groups, it would be intuitive to think it would be efficacious in the Both injury group. Designing the analyses this way allows us to make informative decisions about the Both injury group (e.g., what is the optimal dose that providers should use in practice) without completely disregarding the collected data from the Both group. This design still controls the type I error rate in each injury group under the global null scenario (i.e., no benefit in any injury group).

## Conclusion

The TIC-TOC trial will provide critical information to medical research for the treatment of children with hemorrhage injuries. Existing established trial methods have been incorporated into this novel Bayesian adaptive design to increase the efficiency of the study. The design allows for inferences of optimal design in various types of injuries and allocates more patients to doses that appear to be most beneficial throughout the study. It would not be feasible to study children with multiple trauma consisting of both brain and torso injuries without this design. At the end of the trial, investigators will have better guidance of whether TXA is efficacious for children with hemorrhagic injuries and what dose of TXA to use for the specific injury types.

## Supplementary Information

**Additional file 1.**

## Data Availability

All data in this manuscript were simulated.

## Abbreviations

AUC: Area under the curve
CRASH: Clinical Randomization of an Antifibrinolytic in Significant Hemorrhage
PedsQL: Pediatric Quality of Life Inventory
TIC-TOC: Traumatic Injury Clinical Trial Evaluating Tranexamic Acid in Children
TXA: Tranexamic acid
