# Supplementary material for: The design of a Bayesian adaptive clinical trial of tranexamic acid in severely injured children
Source: Trials. 2021 Nov 4;22:769. doi: 10.1186/s13063-021-05737-0 (PMC8567588; doi:10.1186/s13063-021-05737-0)
Supplement: Supplementary file 1 — Additional file 1. [file 13063_2021_5737_MOESM1_ESM.docx]

# Supplemental Material: Adaptive Design Report

# Introduction

TIC-TOC is an adaptive clinical trial using Bayesian response-adaptive randomization (RAR) to investigate multiple doses of TXA for improving PedsQL outcomes in children having hemorrhagic injuries to either the Brain, Torso, or Both. The trial will investigate up to three doses of TXA (low = 15 mg/kg, mid = 30 mg/kg, high = 45 mg/kg) along with a placebo. The goals of the trial are to evaluate the benefit of TXA relative to placebo for each injury group and to identify the dose of TXA. RAR will be used to efficiently explore the dose range.

## Sample Size

Up to a maximum of 2000 children will be enrolled, but accrual to some or all of the injury groups may be discontinued before the trial reaches the maximum sample size, either due to futility or early expectation of demonstrating benefit.

Additionally, a sample size cap is applied to each injury group. Based on clinical knowledge about the proportions that these injuries occur (assumed to be 60% Brain injuries, 30% Torso injuries, 10% Both injuries), the maximum sample size for the Brain, Torso, and Both groups are set to 1600, 900, and 300 patients, respectively. These caps allow for fluctuation in the assumed injury group distribution since the maximum total sample size stays fixed at 2000 patients. They also mitigate the risk that the enrolled population will be completely dominated by the faster enrolling groups. The caps also help to prevent excessively prolonging the study (for example, if one or more of the injury groups stops early).

## Treatment Arms

Patients will be randomly assigned to one of three doses of TXA (low = 15 mg/kg, mid = 30 mg/kg, high = 45 mg/kg) or to placebo. The high dose will not be available in the initial randomization, but may be opened for investigation later in the trial. The first 500 total patients will be assigned equally to placebo, low and mid doses, stratified by injury group. Afterwards, the allocation probabilities in each injury group will be adjusted after every 250 new patients.

## Primary endpoint

The primary efficacy endpoint for the trial is the Pediatric Quality of Life Inventory (PedsQL) survey, which will be administered at the following times post-injury:

- Week 1
- Month 1
- Month 3*
- Month 6*

Patients with a Torso injury recover more quickly that patients with Brain or Both injuries and thus will only have PedsQL at the Week 1 and Month 1 timepoints used in the primary outcome calculation (asterisks [*] above indicate not used in Torso primary outcome). To capture this aspect of the endpoint, we analyze the longitudinal assessments of PedsQL using area under the curve (AUC), which will be calculated for each patient using the trapezoidal method. The trapezoidal method uses whatever time points have been observed in the follow-up periods and removes missing time points from the calculation.

# Statistical Modeling

## Dose Response Model (for a single injury group)

The hyperbolic $E_{max}$ model utilizes a non-linear function and is commonly used in dose-finding studies. It has been used in many fields and in all stages of drug development (Thomas, Sweeney, & Somayaji, 2014). The model can be defined by

$$R_{i}=E_{0}+\frac{D_{i}\times E_{max}}{D_{i}+ED_{50}}+\varepsilon_{i}$$

where $R_{i}$ is the response or outcome for individual $i$, $E_{0}$ is the average placebo response, $D_{i}$ is the dose for individual $i$, $E_{max}$ is the maximum effect attributable to the drug (i.e., the maximum benefit on your outcome scale you could possibly receive while taking the drug), $ED_{50}$ is the dose that results in half of $E_{max}$, and $\varepsilon_{i}$ is a random error associated with individual $i$ (Macdougall, 2006). The variable $E_{max}$ dictates whether the overall non-linear function has a positive trend or a negative trend. Once the three parameters in the model are estimated, the function can be used to extrapolate the effect sizes of the drug for doses not observed in the model (e.g., estimate the effect of a higher dose not currently being used). This is discussed further in the Opening the High Dose section below. In a Bayesian framework, each parameter is estimated using not only the observed data but also accounting for the prior distributions of the model parameters. Assessing several doses within a dose response model eliminates the multiplicity issue of testing multiple doses because the model estimates the overall dose relationship compared to placebo instead of individual drug dose comparisons. If active drug is determined to be superior to placebo, the parameter estimates allow for determination of optimal dose so providers know what dose to use in practice.

## Dose Response Model (extended to accommodate multiple injury groups)

In the TIC-TOC trial, there will be three distinct, yet related, populations studied (Brain injury, Torso injury, and Both injuries). The hyperbolic $E_{max}$ model is typically used within a single population of interest. We incorporate the three different hyperbolic $E_{max}$ models for the three populations into a single framework and borrow information about the model parameters across these populations using a hierarchical model. That is, the hyperbolic $E_{max}$ is extended to the following:

$$R_{i,k}=E_{0,k}+\frac{D_{i,k}\times E_{max,k}}{D_{i,k}+ED_{50,k}}+\varepsilon_{i,k}$$

where $i$ is a unique patient within injury population $k$ ($k\in(1,2,3)$ for Brain, Torso, and Both, respectively). The similar parameters in each model are assumed to come from common distributions:

$E_{0,k} \sim N\left( \mu_{E_{0}},\sigma_{E_{0}}^{2} \right)$ for $k\in(1,2,3)$

$E_{max,k} \sim N\left( \mu_{E_{max}},\sigma_{E_{max}}^{2} \right)$ for $k\in(1,2,3)$

$\log\left( ED_{50,k} \right)\sim N\left( \mu_{\log\left( ED_{50} \right)},\sigma_{\log\left( ED_{50} \right)}^{2} \right)$for $k\in(1,2,3)$

$$\varepsilon_{i,k}\sim N\left( \mu_{\varepsilon},\sigma_{\varepsilon}^{2} \right)$$

The symbol $\sigma^{2}$ is used to reflect the variance of the respective parameters. In the model, the log of the $ED_{50}$’s is used to allow for the entire real spectrum of numbers to be captured instead of only positive values (Thomas et al., 2014). Although the $\log\left( ED_{50,k} \right)$’s are unrestricted, the $ED_{50,k}$’s will rightly be positive.

This hierarchical structure allows for the integration (“borrowing”) of information between parameters for the three injury groups. The amount of borrowing for each parameter is influenced by the pre-specified hyperprior distributions. As described in Berry, Broglio, Groshen, and Berry (2013), borrowing via a hierarchical model is a type of shrinkage estimation; it provides a formal mechanism by which extreme observations are shrunk toward the overall mean. The shrinkage estimation results in a reduced variance which can increase the precision of the estimates without increasing the sample size.

Each hyperparameter is assigned its own respective hyperprior distribution:

$$\mu_{E_{0}}\sim N\left( 0, 1000 \right)$$

$$\mu_{E_{\max}}\sim N\left( 0,1000 \right)$$

$$\mu_{\log\left( ED_{50} \right)}\sim N\left( 3.2, 1/9 \right)$$

$$\mu_{\varepsilon}\sim N\left( 0, 1000 \right)$$

$$\sigma_{E_{0}}^{2}\sim IG\left( 0.001,0.001 \right)$$

$$\sigma_{E_{\max}}^{2}\sim IG\left( 0.1,19.9 \right)$$

$$\sigma_{\log\left( ED_{50} \right)}^{2}\sim IG\left( 7,0.5 \right)$$

$$\sigma_{\varepsilon}^{2}\sim IG\left( 0.001,0.001 \right)$$

For each dose-response parameter, the set of means (from each injury group) are assumed to come from a common normal $\left( N \right)$ distribution, parameterized by mean and variance, and the variances (1/precision) are assumed to come from a common inverse gamma $\left( IG \right)$ distribution with parameters shape and rate.

The hierarchical priors were calibrated, through simulation, to induce the desired borrowing behavior across the three injury groups. The variance of that common distribution influences how much or how little borrowing takes place across groups. A small variance (high precision) will encourage the parameter estimates to borrow information from each other and converge to a common mean, whereas a large variance (low precision) will allow the parameter estimates to be more heavily influenced by their respective data. Importantly, the estimate of the variance parameter is updated based on the observed outcome data, so that more borrowing occurs when the data are consistent across groups and less borrowing occurs when the data are less consistent across the groups. The phenomenon of borrowing more versus less can be observed in the two example trials below.

The prior distribution for the mean of the $\log\left( ED_{50} \right)$ is mildly informative and was chosen to cover the range of plausible doses for the $ED_{50}$. The $N\left( 3.2, 1/9 \right)$ on the log scale transforms be centered at approximately 25 mg/kg and covers the range of plausible doses between 0 mg/kg to 45 mg/kg. The priors for the variance of $E_{max}$ and $\log\left( ED_{50} \right)$ were chosen to optimize the power of the Both group under various scenarios while keeping the Type I error at an acceptable level.

The Bayesian hierarchical dose-response model is fit to the data at each interim and at the final analysis. The posterior is evaluated using Markov chain Monte Carlo methods using all of the data available at the time of each analysis. Posterior quantities from the model will be used to drive adaptations and decisions during the trial.

## Target Dose

Based on the fit of the dose response model, we identify the 80% effective dose (ED80), defined as the smallest available dose (among 15, 30, or 45 mg/kg) that achieves at least 80% of the estimated treatment effect of the highest dose, relative to placebo. The dose identified as the ED80 may be different among the injury groups. This target dose will be used to guide the RAR algorithm.

## Probabilities for Decision Making

Three key probabilities will be computed and used to make interim and final decisions for the trial. For each injury and each dose, we compute:

- The probability that the dose is the ED80 within the injury group: Pr(d = ED80, k)
- The probability of a positive treatment effect: Pr(Emax,k > 0)
- The probability that the 45 mg/kg dose is at least 4.5 QoL units better than placebo

# Adaptive Design

Adaptive decisions that are evaluated at the interims include the following:

- Discontinuation of enrollment to an injury group, either for futility or for expected success
- Opening the high dose arm within an injury group
- Adjustment of the randomization probabilities within each injury group

All of the planned adaptations are completely prespecified and are described in more detail below.

## Timing of Interim Analyses

The first interim will occur once 500 patients have been randomized. Subsequent interims will be performed after every 250 new patients randomized so that the interims occur after 500, 750, 1000, 1250, 1500, and 1750 patients randomized. Adjustments to RAR probabilities begin at the first interim analysis. Evaluation of early stopping decisions will begin at the third interim (after 1000 total patients are enrolled). Only individuals who have an observed outcome will contribute to the response adaptive randomization. This means that recently randomized patients without an observed outcome will not contribute when the study hits the interim sample size (e.g., 500), but will contribute their information to the following interim assuming they have an observed outcome at that point. Because this study is implemented in a blinded fashion and study drug is given over 8 hours, patients who were randomized to an arm that is dropped will have completed treatment by the time the allocations are updated and will not be notified of the arm dropping.

## Response-adaptive randomization

The trial will begin with an initial “burn-in” period for the first 500 total patients, in which the patients within each injury group will be randomized in equal proportion to placebo, the low dose of TXA and the mid dose of TXA (33% probability to each, and 0% probability to the high dose).

After this initial burn-in period, the randomization probabilities will be updated after every 250 new patients randomized, with the goal of preferentially allocating patients to the doses with the highest chances of being the ED80.

Allocation to the placebo arm within each injury group will be fixed at 33% throughout the trial (with one exception as described below). The remaining 67% probability will be distributed among the TXA doses in proportion to the probability that each dose is the ED80, Pr(d = ED80). The probabilities will be normalized to sum to 100%.

The following additional constraints on RAR will be applied within each injury group:

- In order to avoid assigning patients to a dose with minimal chance of being the ED80, we set the randomization probability to zero for any dose with allocation probability less than 10%. The probability is reallocated among the remaining doses and renormalized to sum to one. In this way, a dose may be temporarily dropped for an injury group, but may be re-introduced if the adaptive randomization probability increases at subsequent interims.
- If only one TXA dose remains after the previous bullet point, then patients will be randomized equally, 50% probability to the placebo and the one TXA dose.

## Opening the High Dose

Beginning at the first interim at 500 patients randomized, we will evaluate whether the 45 mg/kg dose should be introduced within each injury group. The following conditions must both be satisfied in order to add the high dose:

- There is at least a 50% posterior probability that the model-estimated difference in AUC between the 45 mg/kg dose and placebo is greater than or equal to 4.5 QoL units for that injury group (the minimum clinically meaningful difference), and
- There are no safety concerns for a higher dose identified by the Data and Safety Monitoring Board based on current data.

If the 45 mg/kg dosing arm is opened within an injury group, the initial randomization probability for that dose will be capped at no more than 20% until the probabilities are re-evaluated at the next interim look. This cap is instituted to ensure that preliminary safety data can be collected on the 45 mg/kg arm within an injury group before a larger proportion of patients are randomized to that dose. After the 45 mg/kg dose has been opened up for at least one interim period, the maximum allocation proportion for the 45 mg/kg dose increases to 50%.

## Stopping an Injury group for Futility or Expected Success

This design has prospectively defined stopping rules that allow accrual to an injury group to be stopped once the question of benefit has been answered for that group. Early stopping decisions are evaluated starting at the third interim (1000 total patients randomized). An injury group, k, may be stopped:

- For reaching a sample size cap (1600, 900, and 300 patients, respectively for the Brain, Torso, and Both groups)
- For futility if the posterior probability of a positive treatment effect, Pr(Emax,k > 0), is sufficiently low for injury group k
- For expected success if the posterior probability of a positive treatment effect is sufficiently high.

The stopping boundaries for futility and expected success vary by injury group and by interim, as shown in Table 1. These boundaries were carefully calibrated through simulations. The thresholds were selected to control the type I error rate at 2.5% (one-sided) within each group under the scenario that assumes no benefit in any group. See the Operating Characteristics section for more detail.

Table 1: Boundaries of posterior probabilities of efficacy for stopping accrual and for final analysis decisions

| Total Sample Size | Brain | | Torso | | Both | |
| --- | --- | --- | --- | --- | --- | --- |
|  | Efficacy | Futility | Efficacy | Futility | Efficacy | Futility |
| 1000 | 0.990 | 0.050 | 0.980 | 0.050 | 0.975 | 0.050 |
| 1250 | 0.990 | 0.100 | 0.980 | 0.100 | 0.975 | 0.100 |
| 1500 | 0.990 | 0.150 | 0.980 | 0.150 | 0.975 | 0.150 |
| 1750 | 0.990 | 0.200 | 0.980 | 0.200 | 0.975 | 0.200 |
| Final Analysis | 0.981 | 0.250 | 0.978 | 0.250 | 0.975 | 0.250 |

When a stopping boundary is crossed, recruitment is stopped for that injury group, and the patients currently enrolled in that injury group continue to have follow-up until all individuals in that group have been evaluated at all follow-up time periods.

Due to rarity of patients with Both injuries, if both the Brain and Torso injury groups stop enrollment for any reason, we will stop recruitment in the Both injuries group regardless of what the current effect size is. This is to prevent the trial from continuing for an infeasible amount of time.

## Final Analysis

The final analysis for an injury group will occur once all patients in that group have completed follow-up. Thus, while accrual to a group may end before the maximum sample size, there is only one decisive analysis to evaluate success within the group. The posterior probability of a positive treatment effect (i.e, Pr($E_{max}$>0)) once all data are collected is compared to the injury group’s final pre-specified threshold for success. This is similar to how other analyses have evaluated efficacy in the past with $E_{max}$ models (Gajewski et al., 2019). The final analysis will use whatever data are available at the time in the other injury groups due to the hierarchical model.

### Final Analysis of the Both Group

Due to the lower prevalence of children with Both injuries, we do not expect that the trial will have sufficient power at a traditional level (e.g., 80%) to detect benefit in this group on its own mid-trial, even with the hierarchical component. At the end of the trial, if both the Brain and Torso groups are shown to be efficacious and the Both group has not yet crossed a stopping boundary, a separate hyperbolic $E_{max}$ model will be performed on the Both injury group. For this model, the prior for each dose-response parameter ($E_{0}, E_{max}, \log\left( ED_{50} \right)$) will have the mean value set equal to the average of the parameter estimates from the Brain and Torso groups. The prior variance will be increased to 150% of the average variance estimate for each respective parameter across the Brain and Torso groups. This is done so the isolated injury groups do not completely inform the inference for the Both group.

# Example Trials

Two different example trials are presented to demonstrate the various adaptations incorporated in the design. Within each trial, a summary page is presented for each interim look (starting at 500 patients).

On the interim summary page (Figure 1), the dose-response curve estimates compared to the placebo (solid line) along with the 95% credible interval (shaded area) are displayed for each injury group (shown in the first column of plots in the green box below). Raw observed means for the injury group arm are represented by a red dot. If there is no red dot at the 45 mg/kg dose, that indicates there is no observed outcome data yet in that injury group arm. Values above 0 indicate TXA is more efficacious than placebo, while values less than 0 indicate TXA is more harmful than placebo.

For each injury group and arm, the total number of patients enrolled, the proportion of patients randomized to the arm, and the number with complete follow-up data are displayed in a table (shown in the middle column of tables with a blue box in Figure 1). The new allocation proportions based on the observed data are also displayed.

The posterior probability of efficacy and the current interim stopping boundaries (efficacy and futility) are displayed in a table (shown in the right column of tables in the red box below). When the posterior probability of efficacy is larger/smaller than the efficacy/futility threshold, the injury group stops enrollment and declares TXA efficacious/futile, respectively.


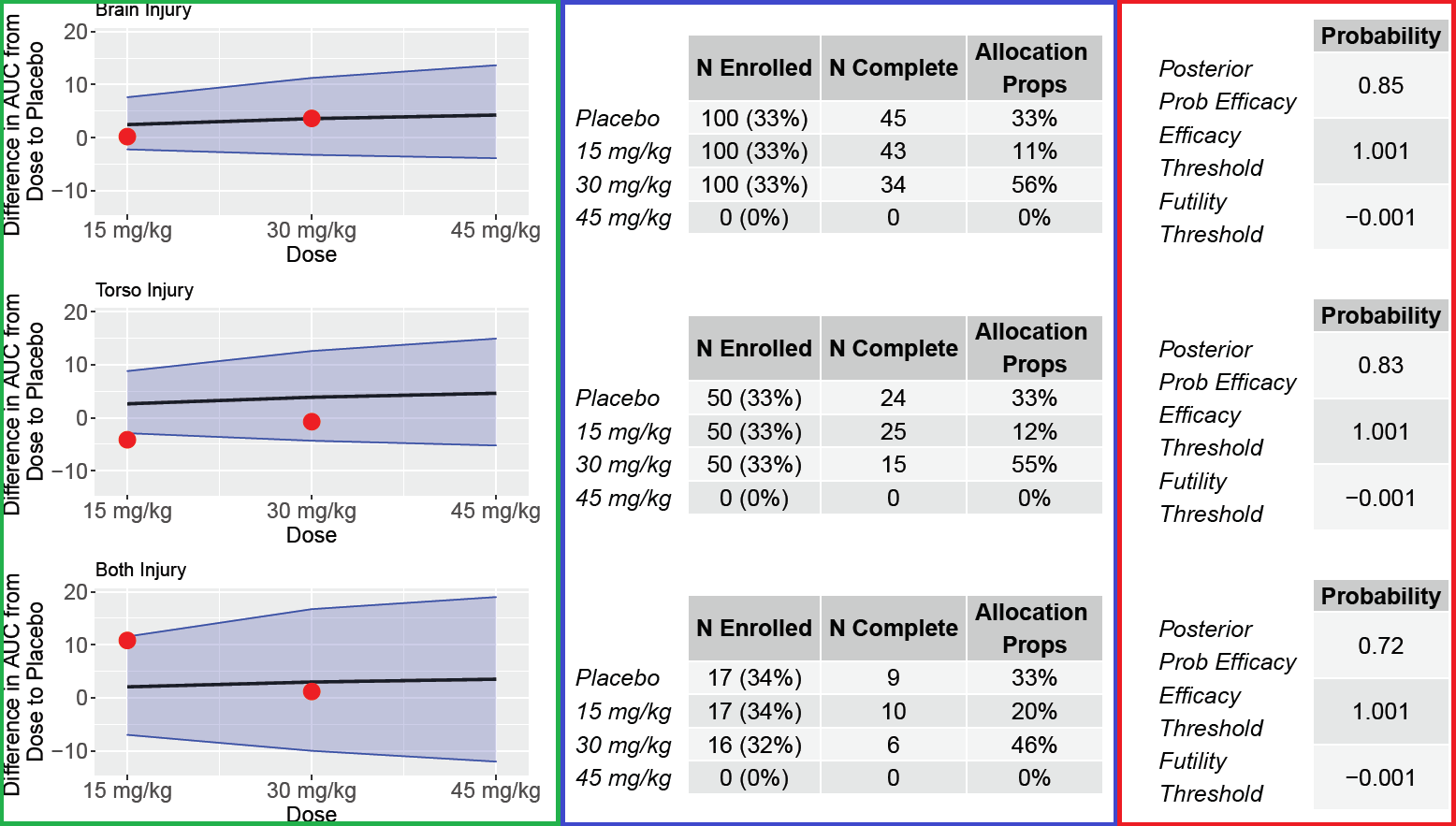


Figure 1: Interim data presented from an example trial

## Example Trial 1

The first example trial assumes an average monthly enrollment rate of 30 children with Brain injuries, 15 with Torso injuries, and 5 with Both injuries per month. This simulation is simulated from Scenario 3 (quantified in Table 2 in the Simulation Scenarios below). At the first interim look of 500 patients (Figure 2.1), approximately half of the enrolled patients have an outcome observed. The Brain injuries group has 122 observed outcomes (45 placebo, 43 at 15 mg/kg, 34 at 30 mg/kg), whereas the Torso injuries group has 64 observed outcomes and the Both injuries only group has 25 observed outcomes. At this first interim look, we see a positive estimated relationship between TXA dose and change in AUC compared to placebo in the Brain and Torso groups (as depicted by the solid black line). These positive relationships are driving the overall relationship in the Both group due to the limited data available in the Both group even though observe a negative relationship in the Both injury group. There is a wide credible interval estimated in the Both injury group due to this conflicting information. This wide credible interval accurately portrays the range of plausible effects that could be observed based on the given data.

Based on the dose response curve, the allocation in the 30 mg/kg arms are increased for the next set of 250 enrolled patients for each injury group. The placebo arm stays fixed at 33%. At this current interim, the posterior probability that TXA is efficacious in the Brain, Torso, and Both injury groups are 0.85, 0.83, and 0.72, respectively, but the specified boundaries do not allow for stopping. The trial continues enrolling all three types of injury. Formal efficacy and futility monitoring do not occur yet as indicated by the unachievable efficacy and futility thresholds (set to 1.001 and –0.001, respectively, to effectively disable stopping of accrual at the interims with N<1000).


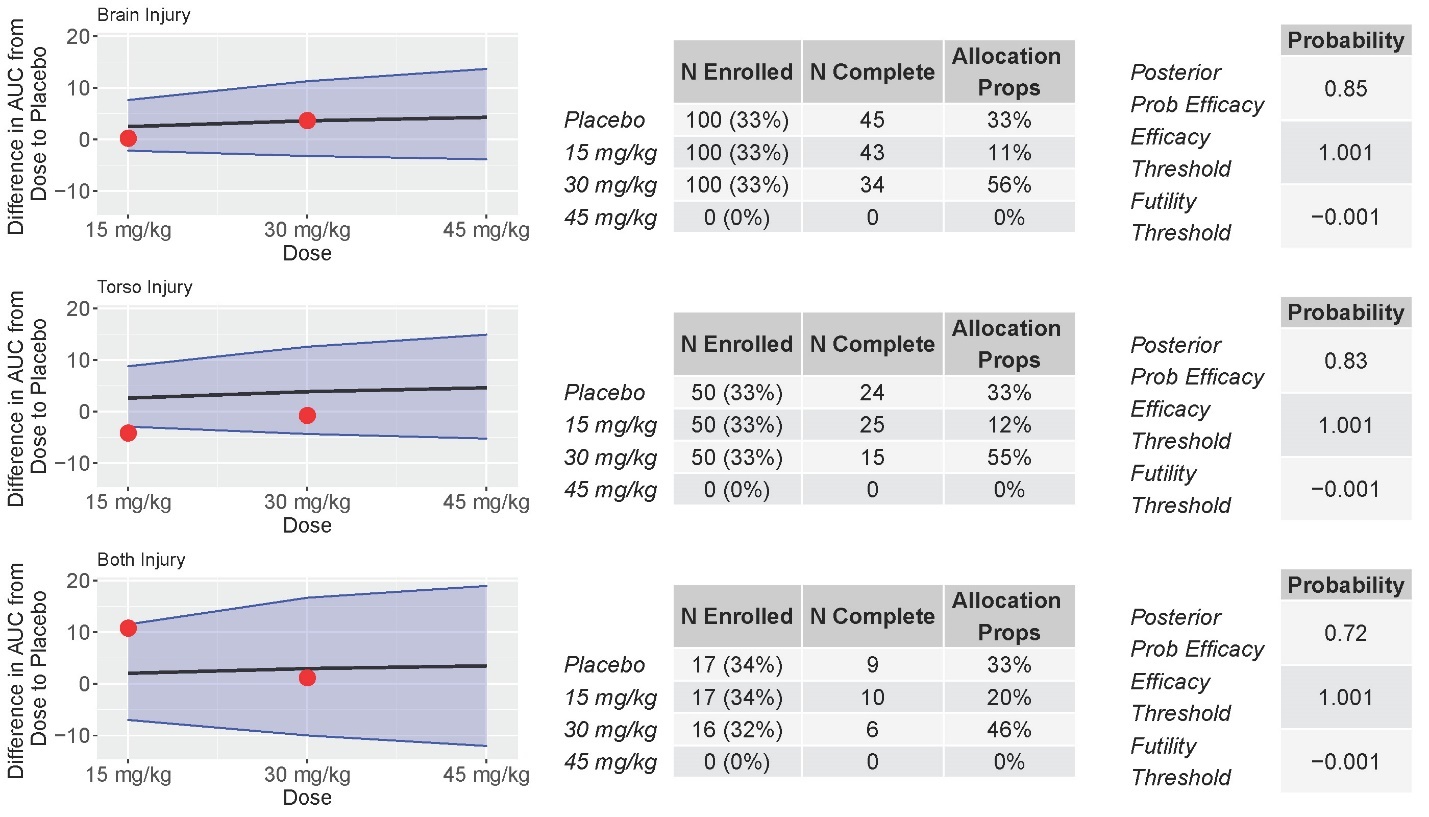


Figure 2.1: Interim data (n=500) from Example Trial 1

After an additional 250 patients have been enrolled (n=750), we are still seeing a positive relationship in TXA dose and change in the AUC for the Brain and Torso injuries group (Figure 2.2). In the Brain injury group, the 15 mg/kg dose allocation proportion fell less than 10%. This dose in the Brain injury group is temporarily dropped from the randomization sequence until the next interim look (at which point will be re-evaluated). The Torso group estimate is relatively flat and all three doses are equally allocated in this injury group. The Both group dose-response curve is still driven by the Brain injury group due to the smaller sample size. For this reason, the allocation proportions are still favoring the 30 mg/kg arm. An additional 250 patients will be enrolled until the next interim look.


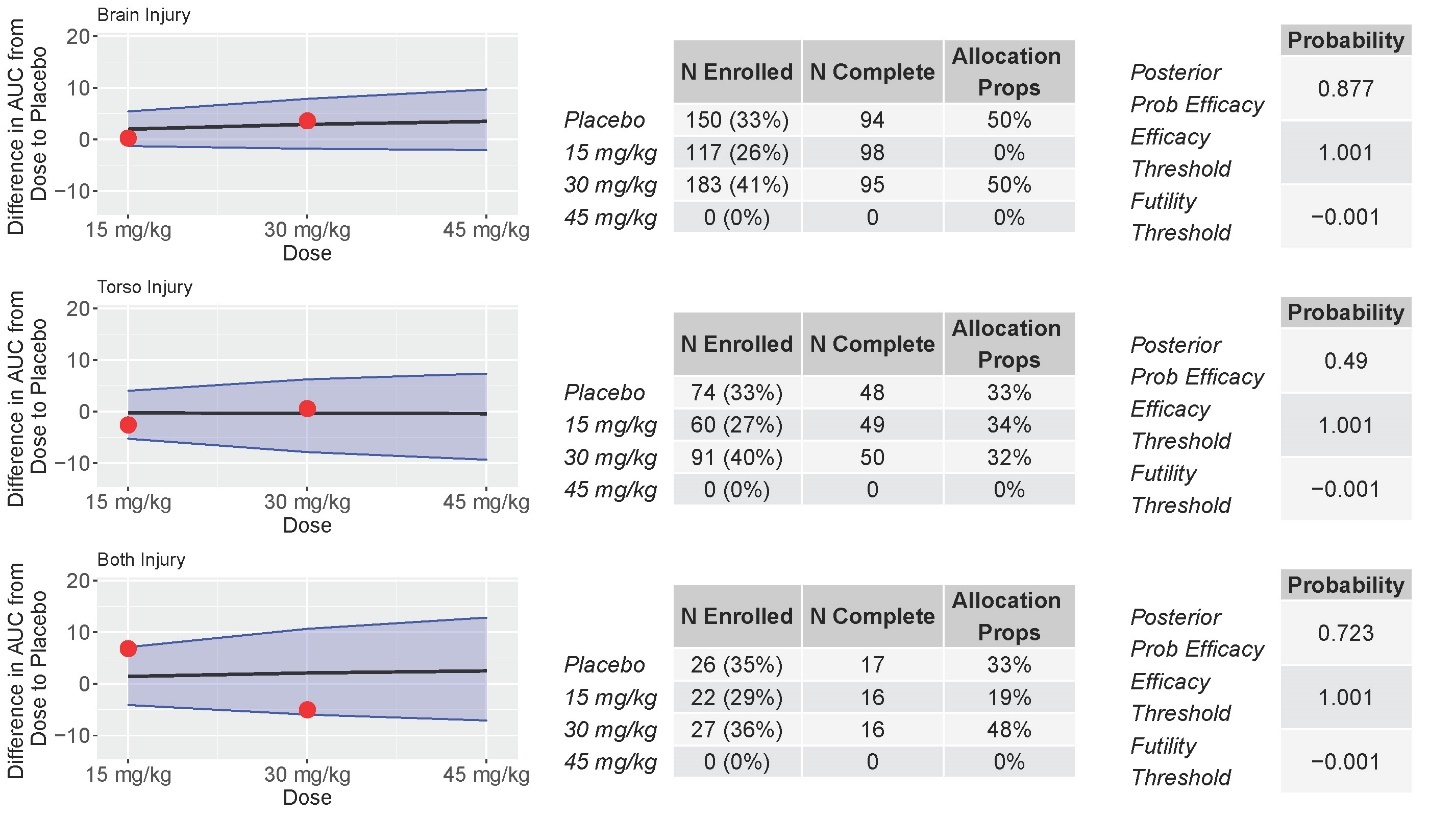


Figure 2.2: Interim data (n=750) from Example Trial 1

Efficacy and futility monitoring will begin after 1000 patients have been enrolled. At this interim look (Figure 2.3), we still observe an upward slope for the dose response curves in the Brain and Torso injuries groups. The Both injury group now has observed additional outcomes and now has a slight downward slope. The Brain and Torso injuries have a higher allocation proportion to the 30 mg/kg arm compared to the 15 mg/kg arm for the next 250 enrolled patients. Due to the estimated downward dose-response curve in the Both injury group, more patients are randomized to the 15 mg/kg arm compared to the 30 mg/kg arm. The efficacy thresholds for the Brain, Torso, and Both injuries groups are 0.99,0.98, and 0.975, respectively. The posterior probability of efficacy in the Brain injury group (0.92) approaches the boundary, but the trial continues enrollment because no posterior probabilities cross their respective boundaries.


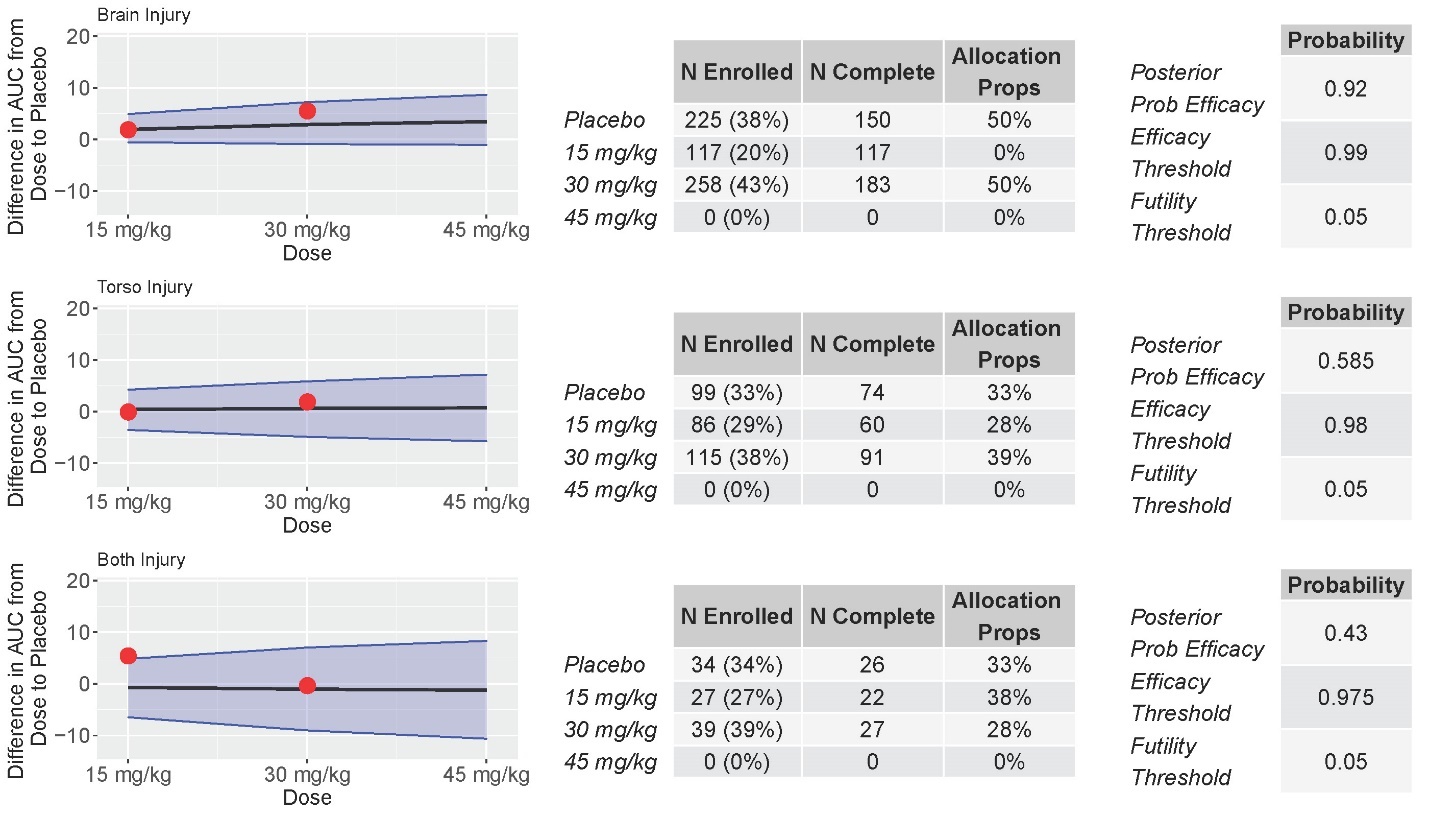


Figure 2.3: Interim data (n=1000) from Example Trial 1

As seen in Figure 2.4, after 1250 patients were enrolled, the efficacy threshold is crossed in the Brain injury group (observed posterior probability of success is 0.998; threshold for efficacy is 0.99). The study stops enrolling in the Brain injury group which is demonstrated by the 0% allocation proportions for this injury. The other two injury groups proceed with enrollment.


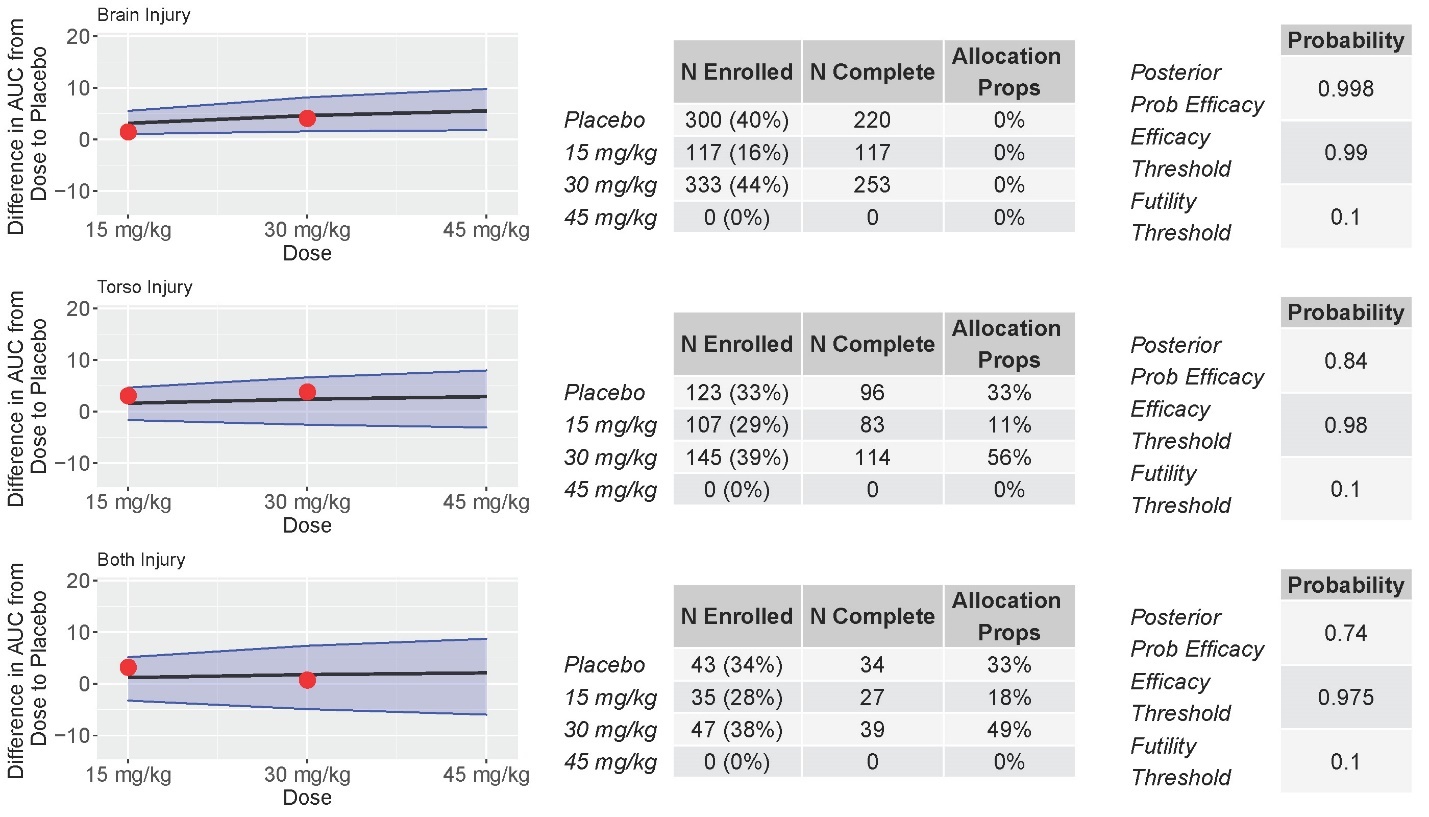


Figure 2.4: Interim data (n=1250) from Example Trial 1

After 1500 patients had been enrolled, we observe that the Brain injury group now has 100% observed follow-up (Figure 2.5). The Torso injury group crossed the threshold for efficacy so enrollment is stopped in the Torso injury group. Due to enrollment stopping in both the Brain and Torso injury groups, enrollment is stopped in the Both group to avoid an infeasible amount of time. Because the Brain and Torso group crossed the efficacy boundary, the Both group will be re-analyzed once all follow-up is complete using the updated priors observed based on data in the Brain and Torso groups.
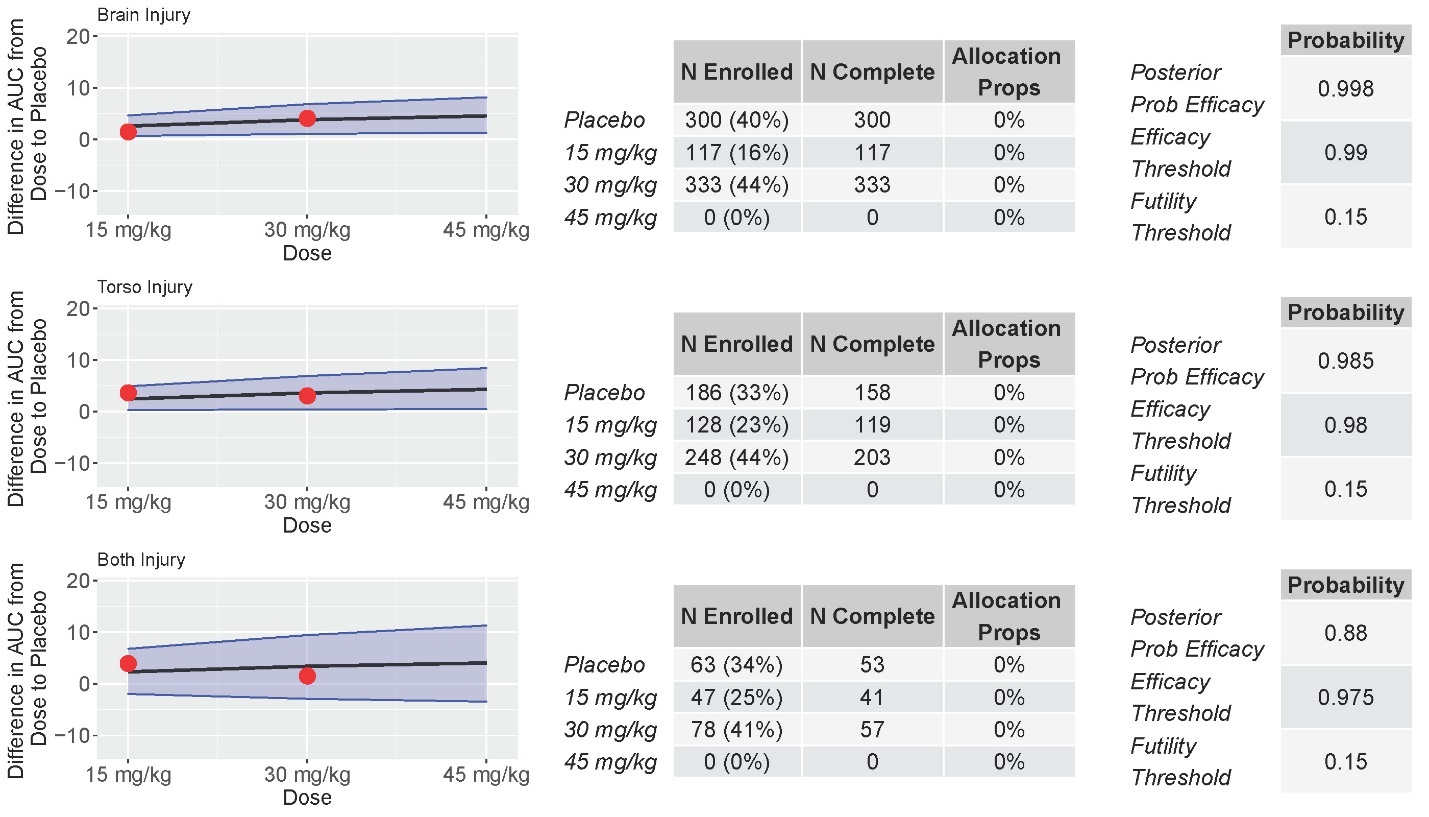


Figure 2.5: Interim data (n=1500) from Example Trial 1

## Example Trial 2

The second example trial assumes the same enrollment rates as the first example trial. This simulation is simulated from Scenario 9 (quantified in Table 2 in the Simulation Scenarios below). At the first interim look (Figure 3.1), there is an estimated positive effect between TXA compared to placebo in the Torso injury group and the Both injury group, but the Brain injury group has an observed negative relationship. Due to the magnitude of estimated relationship in the dose response curve, the posterior probability of success is larger in the Torso and Both groups and the 45 mg/kg arm opens in these injury groups. The 15 mg/kg arms are temporarily dropped from the allocation sequence for these two injury groups. The dose response curve in the Brain injury group results in the 30 mg/kg arm being temporarily dropped from the allocation and all patients will temporarily be randomized equally to the placebo and 15 mg/kg arm. Efficacy and futility monitoring does not begin until 1000 patients are randomized.


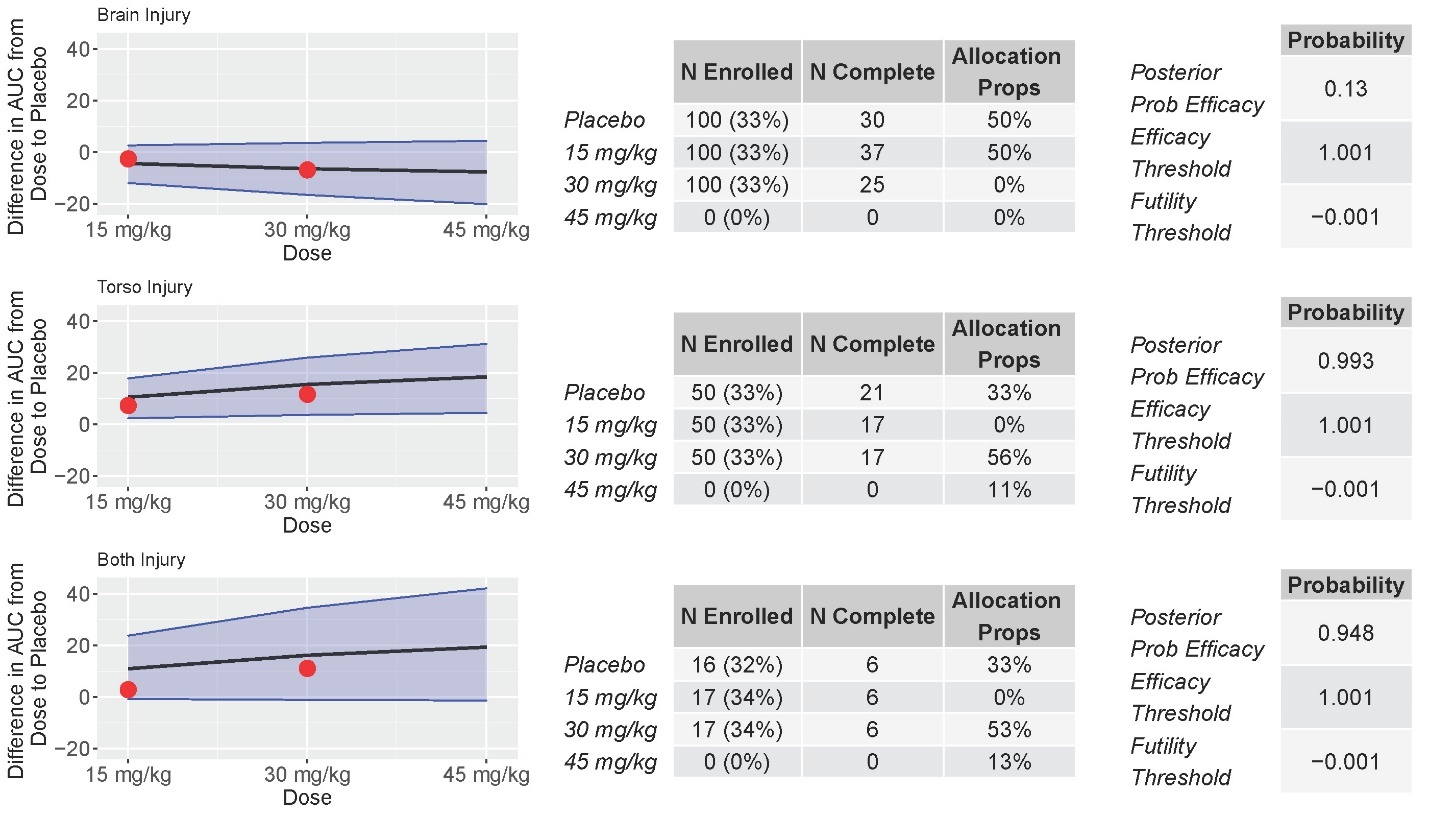


Figure 3.1: Interim data (n=500) from Example Trial 2

After an additional 250 patients are enrolled (n=750), we observe a downward dose-response curve in the Brain injury group and an upward dose-response curve in the Torso and Both injury groups (Figure 3.2). Due to the negative relationship of TXA dose and the PedsQL AUC, the 30 mg/kg arm remains temporarily dropped in the Brain injury group. In the Torso and Both injury groups, the 45 mg/kg arm (which was added at the last interim) is now temporarily dropped based on the accumulating data.


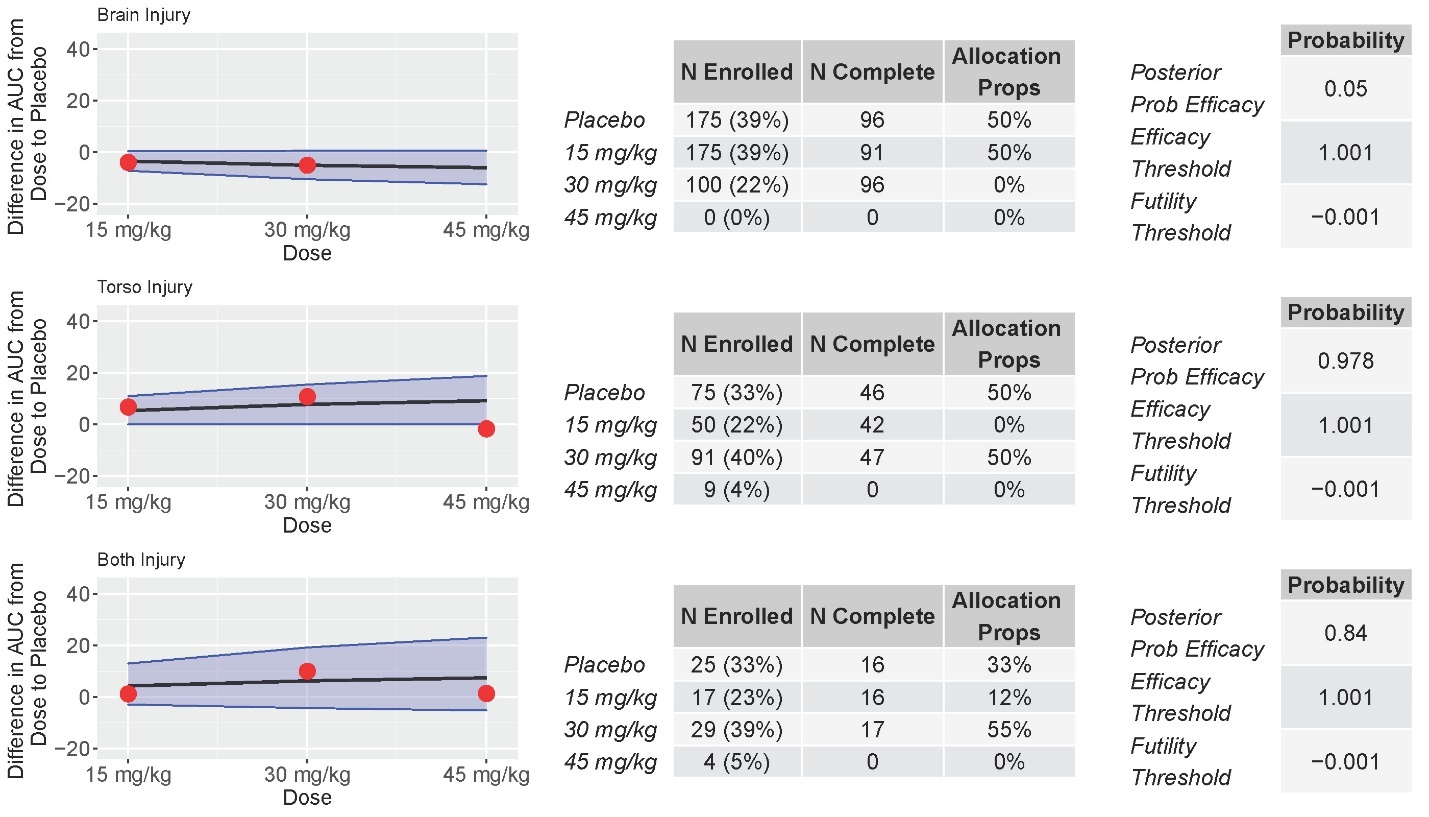


Figure 3.2: Interim data (n=750) from Example Trial 2

At the n=1000 interim look, we begin evaluating for efficacy and futility. At this interim, the posterior probability of efficacy in the Brain injury group is 0.032 which is less than the futility threshold of 0.05. Enrollment ceases in the Brain injury group. The Torso injury group has an observed posterior probability of efficacy (0.998) larger than the Torso interim threshold (0.98) so enrollment ends in the Torso injury group for anticipated efficacy. The formal efficacy look will occur once all patients in the Torso injury group complete follow-up. Due to the stopping of both the Brain and Torso injury groups, the Both injury group is forced to end to not prolong the study for an infeasible time.


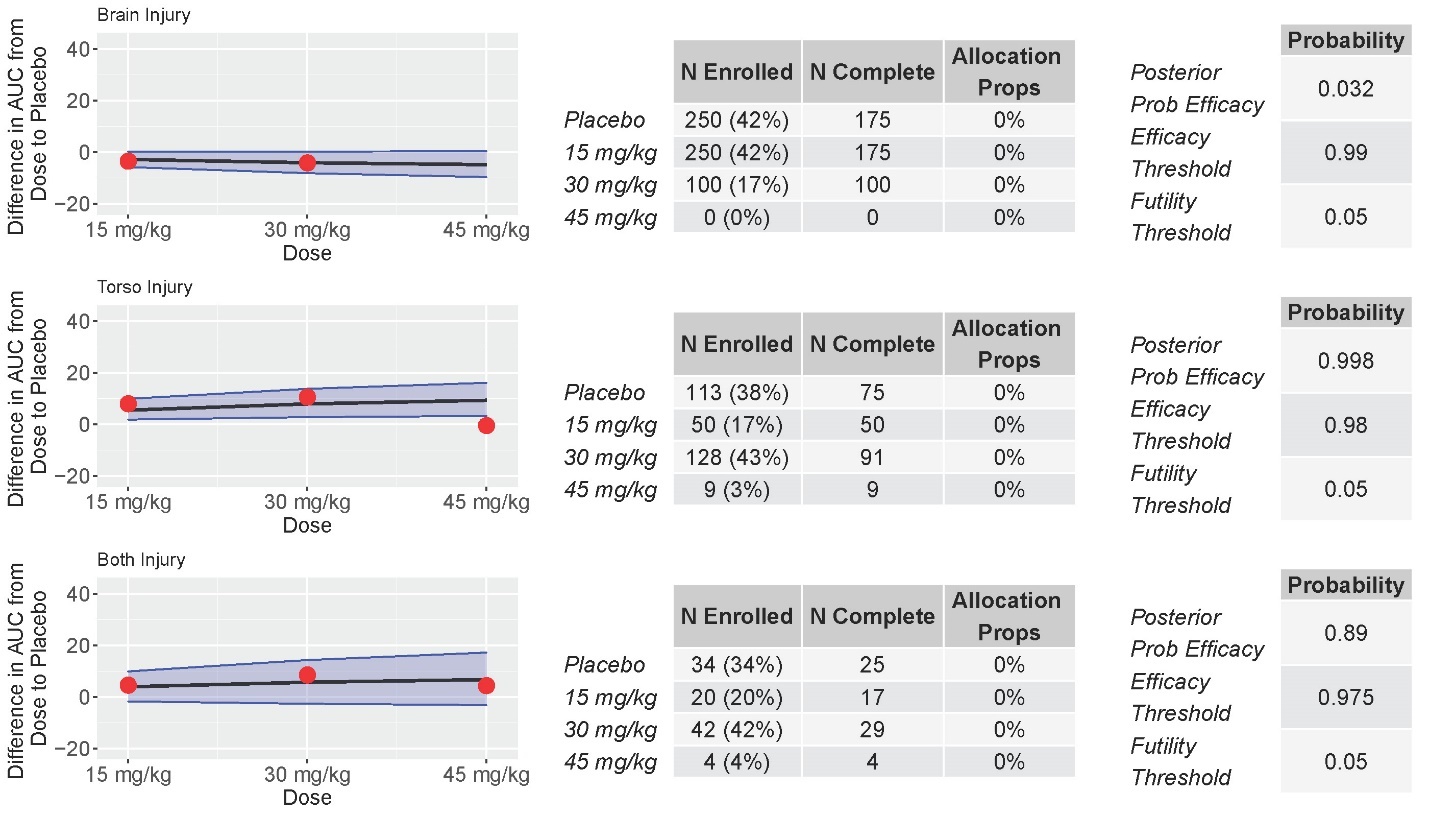


Figure 3.3: Interim data (n=1000) from Example Trial 2

# Simulation scenarios

We evaluate the trial design through computer simulation. We simulated the entire trial multiple times under various hypothesized scenarios for the underlying truth. In each simulated trial, interim analyses were conducted according to the pre-specified rules and the results were recorded for each trial. This section describes the parameters that were used to generate the data for the simulations.

## Accrual

We simulated patients being enrolled in the trial at a rate of 50 per month (30 Brain, 15 Torso, and 5 Both). The simulations assume no lost to follow-up for the primary outcome.

## Prevalence of Injury Groups

We assume in the simulations that the underlying prevalence for the injury groups are 60% Brain injuries, 30% Torso injuries, 10% Both injuries.

## PedsQL AUC Outcomes

We consider nine scenarios for the underlying dose-response relationship. These scenarios are shown in Table 2 and plotted in Figure 4. When simulating data, the natural variability of the AUC around the dose-response curve was assumed to be ${20}^{2}$ which was based on unpublished historical data collected in a different study using PedsQL. The $E_{0}$ (average placebo response) for the Brain, Torso, and Both injury groups were estimated by the study investigators to be 78.8, 58.95, and 56, respectively. In the simulations, outcomes are set to missing until six-months have passed in order to reflect the delay that would be observed in the actual trial. Under the alternative hypothesis used to power the study, we assumed a common $E_{max}$ of 6 and a $ED_{50}$ of 10 (Scenario 2). These parameters correspond to a difference of 3.6 QoL units in AUC of the PedsQL between the 15 mg/kg dose and placebo, 4.5 QoL unit difference in AUC between the 30 mg/kg dose and placebo, and a 4.9 QoL unit difference in AUC between the 45 mg/kg dose and placebo. This effect is assumed to be the same across all three injury groups. Scenarios 3 and 4 represent varying efficacious effect of TXA across the three injury groups.

Scenarios 5 through 8 represent “mixed” scenarios in which there is a treatment benefit in at least one group, and no benefit in at least one group. These scenarios were used to understand the influence of the hierarchical model on study conclusions. Scenario 9 represents a scenario where TXA is harmful in one injury group (Brain) but efficacious in the other two injury groups (Torso and Both).

Table 2: Differences in PedsQL AUC between dose levels and placebo

| Scenario | Brain | | | Torso | | | Both | | |
| --- | --- | --- | --- | --- | --- | --- | --- | --- | --- |
|  | 15 mg/kg | 30 mg/kg | 45 mg/kg | 15 mg/kg | 30 mg/kg | 45 mg/kg | 15 mg/kg | 30 mg/kg | 45 mg/kg |
| 1 | 0.0 | 0.0 | 0.0 | 0.0 | 0.0 | 0.0 | 0.0 | 0.0 | 0.0 |
| 2 | 3.6 | 4.5 | 4.9 | 3.6 | 4.5 | 4.9 | 3.6 | 4.5 | 4.9 |
| 3 | 2.7 | 4.3 | 5.3 | 3.0 | 3.4 | 3.6 | 2.7 | 4.0 | 4.8 |
| 4 | 3.3 | 5.0 | 6.0 | 3.3 | 5.0 | 6.0 | 3.3 | 5.0 | 6.0 |
| 5 | 3.6 | 4.5 | 4.9 | 0.0 | 0.0 | 0.0 | 3.6 | 4.5 | 4.9 |
| 6 | 0.0 | 0.0 | 0.0 | 3.6 | 4.5 | 4.9 | 3.6 | 4.5 | 4.9 |
| 7 | 0.0 | 0.0 | 0.0 | 3.6 | 4.5 | 4.9 | 0.0 | 0.0 | 0.0 |
| 8 | 3.6 | 4.5 | 4.9 | 0.0 | 0.0 | 0.0 | 0.0 | 0.0 | 0.0 |
| 9 | -3.6 | -4.5 | -4.9 | 3.6 | 4.5 | 4.9 | 3.6 | 4.5 | 4.9 |


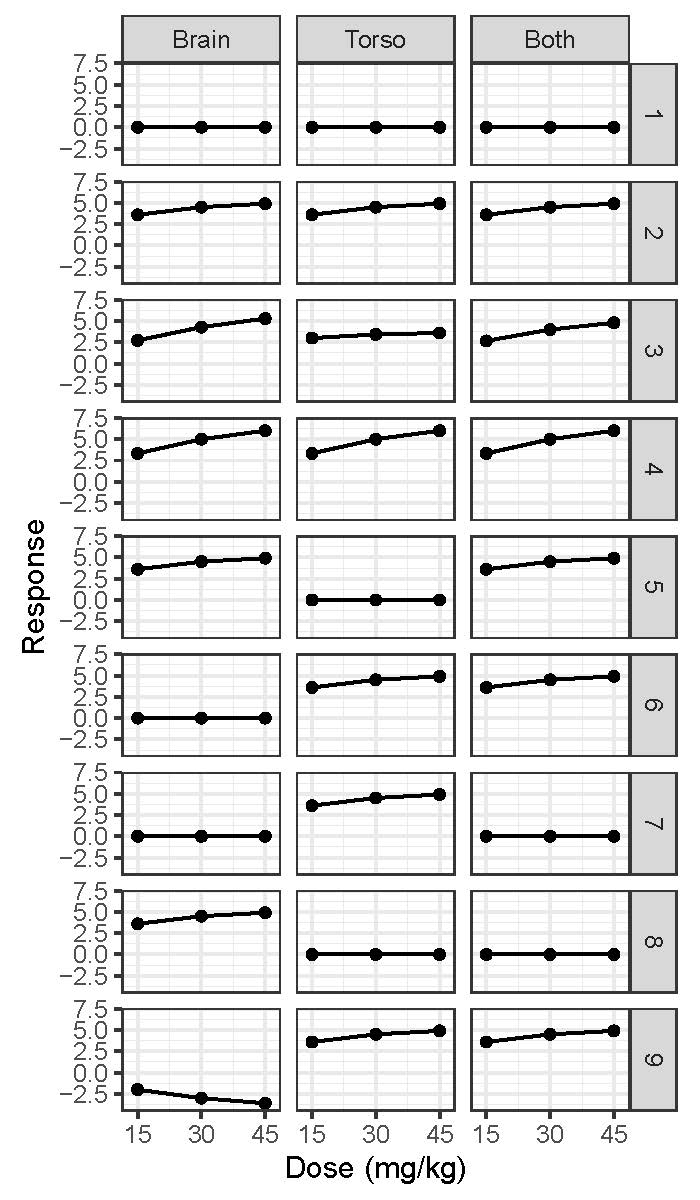


Figure 4: Nine scenarios are presented to understand the operating characteristics of the model. Effect sizes for the PedsQL AUC compared to the placebo arm are plotted for each dose for the Brain, Torso, and Both injury groups. Lines above zero indicate efficacious scenarios where flat lines at zero indicate the null scenario (no benefit).

# Operating Characteristics

## Overall Design Performance

Table 3 shows the overall operating characteristics for each scenario, including the probability of declaring success, the probability of stopping early, and the probability of opening the high dose. We define power in the typical sense of rejecting the null hypothesis of no effect when the null is false. In this trial, within an injury profile, power means successfully identifying TXA is superior to placebo when it is truly superior. When evaluating whether TXA is efficacious compared to placebo, we determine if the probability that the overall dose response curve within the injury profile is superior to placebo is greater than a pre-specified threshold that controls Type I Error (i.e., we are not evaluating each dose of TXA separately compared to placebo). We are estimating the dose response curve through a Bayesian framework. If TXA is found to be superior to placebo, the dose-response curve provides a mechanism to identify the optimal dosage of TXA to give to future injured patients based on the estimated dose-response curve of the enrolled patient population.

Table 3: Operating characteristics of eight different scenarios.

| Scenario | Power | | | Probability of Stopping the Entire  Trial at Specific Interim Looks | | | | | Probability of Opening 45mg/kg Dose | | |
| --- | --- | --- | --- | --- | --- | --- | --- | --- | --- | --- | --- |
|  | Brain^†^ | Torso^†^ | Both^†^ | 1000 | 1250 | 1500 | 1750 | 2000 | Brain | Torso | Both |
| 1 | 2.1% | 2.2% | 1.4% | 0.3% | 1.8% | 2.5% | 4.4% | 91.0% | 21.5% | 20.6% | 21.7% |
| 2 | 94.9% | 82.9% | 64.4% | 16.9% | 22.5% | 19.8% | 15.9% | 24.8% | 73.9% | 70.7% | 63.9% |
| 3 | 92.3% | 64.6% | 52.4% | 10.1% | 15.2% | 15.9% | 16.7% | 42.1% | 72.5% | 65.0% | 65.1% |
| 4 | 97.0% | 89.0% | 73.1% | 23.8% | 27.8% | 20.3% | 14.3% | 13.7% | 79.3% | 76.1% | 68.7% |
| 5 | 92.5% | 5.0% | 39.0% | 2.1% | 4.2% | 5.6% | 18.2% | 69.9% | 67.3% | 35.5% | 59.8% |
| 6 | 4.4% | 65.9% | 30.5% | 0.9% | 2.4% | 4.6% | 5.4% | 86.6% | 30.4% | 63.4% | 56.4% |
| 7 | 3.2% | 61.0% | 3.2% | 0.9% | 2.2% | 4.2% | 5.9% | 86.9% | 27.0% | 57.7% | 30.6% |
| 8 | 90.6% | 4.3% | 5.3% | 1.9% | 4.0% | 5.6% | 11.4% | 77.2% | 64.7% | 31.6% | 34.7% |
| 9 | 0.0% | 71.4% | 24.7% | 3.5% | 11.6% | 18.6% | 20.1% | 46.1% | 9.4% | 53.0% | 41.2% |

^†^ Cells highlighted in red indicate the one-sided Type I error rate in those scenarios. Cells are highlighted in white in an effect and green in the postulated scenario.

Under the global null scenario (Scenario 1), the type-I error rate for all groups is 2.5% or less, and 9.0% of trials stop before the maximum total sample size. There is approximately a 20% chance in each injury group that the 45 mg/kg arm will be opened. Under the hypothesized effect (Scenario 2), the probability of stopping at each interim look ranges between approximately 15% and approximately 25%. There is a 64% to 74% probability in each injury group that the 45 mg/kg dose will be opened.

The probabilities of stopping at each interim look by each injury group are shown in Table 4. In the setting where TXA is more efficacious than hypothesized (scenario 4), we have a high probability of stopping the trial at one of the first efficacy/futility monitoring interim looks. In the mixed scenarios (Scenarios 5-8), we observe there is low probability of stopping early for the injury groups that have the null effect, but there is a higher probability of stopping at earlier interims for efficacious injuries. In Scenario 9 where TXA is harmful in Brain but efficacious in Torso and Both groups, the Brain group tends to stop earlier in the trial while the Torso and Both groups tend to stop more at the maximum sample size in order for the model to learn as much information as possible on the estimated dose-response curve.

Table 4: Proportion of simulated trials that stop at each interim within an injury group

| **Scenario** | **Brain** | | | | | **Torso** | | | | | **Both** | | | | |
| --- | --- | --- | --- | --- | --- | --- | --- | --- | --- | --- | --- | --- | --- | --- | --- |
|  | **1000** | **1250** | **1500** | **1750** | **2000** | **1000** | **1250** | **1500** | **1750** | **2000** | **1000** | **1250** | **1500** | **1750** | **2000** |
| **1** | 3.9% | 5.8% | 6.8% | 7.2% | 76.3% | 4.6% | 6.3% | 6.8% | 8.7% | 73.5% | 2.5% | 5.9% | 7.6% | 9.6% | 74.4% |
| **2** | 45.0% | 19.5% | 13.8% | 9.3% | 12.3% | 30.9% | 21.2% | 16.4% | 12.8% | 18.7% | 26.0% | 23.8% | 18.5% | 13.2% | 18.4% |
| **3** | 38.5% | 19.4% | 14.6% | 10.6% | 17.0% | 19.9% | 15.1% | 14.0% | 14.4% | 36.6% | 17.1% | 19.2% | 16.9% | 13.2% | 33.6% |
| **4** | 54.2% | 20.6% | 12.3% | 6.6% | 6.3% | 36.7% | 25.0% | 16.4% | 11.8% | 10.0% | 33.2% | 27.9% | 18.7% | 10.3% | 9.9% |
| **5** | 38.4% | 19.6% | 14.2% | 10.1% | 17.6% | 4.6% | 4.8% | 5.9% | 17.9% | 66.7% | 10.2% | 11.0% | 13.2% | 12.7% | 52.9% |
| **6** | 3.9% | 4.1% | 5.8% | 5.8% | 80.4% | 21.6% | 14.0% | 11.7% | 11.5% | 41.2% | 8.0% | 7.8% | 9.6% | 10.6% | 64.0% |
| **7** | 4.0% | 4.5% | 6.0% | 6.2% | 79.3% | 17.7% | 13.3% | 11.7% | 11.3% | 46.0% | 2.9% | 5.3% | 8.7% | 10.1% | 73.1% |
| **8** | 35.1% | 19.0% | 13.9% | 10.3% | 21.7% | 4.5% | 5.5% | 6.4% | 11.0% | 72.6% | 4.2% | 7.7% | 10.1% | 11.5% | 66.6% |
| **9** | 21.6% | 23.5% | 19.3% | 15.2% | 20.4% | 17.0% | 14.4% | 17.5% | 15.4% | 35.6% | 8.1% | 15.7% | 20.0% | 18.4% | 37.8% |

In each simulation, the reason for stopping within an injury group is recorded. Table 5 displays the various reasons each injury group was stopped by scenario. In the table, reasons for stopping include reaching the maximum sample size (Reached N=2000), crossing an efficacy boundary anticipating efficacy (Anticipated Efficacy), crossing a futility boundary (Futility Boundary), reaching the maximum sample size allowed for the injury group (Max Injury N), and stopping the Both injury group due to stopping the Brain and Torso injury groups halting enrollment (Forced Close). In this table, when a trial goes to the full sample size (N=2000), trials that cross the efficacy or futility boundary, reach the maximum sample size for the injury group, or end the Both group due to ending the Brain and Torso groups are labeled under their respective columns instead of being categorized under the ‘Reached N=2000’ column if those requirements are met.

In the null scenario (Scenario 1), each group stops between 60% to 70% of the simulations due to hitting the maximum sample size (Reached N=2000) without satisfying other criteria (e.g., anticipated early efficacy). Even though there is a larger probability of a single injury group stopping for futility (between 24.5% and 30.1%), the overall trial continues to the full sample size across all simulations approximately 91% of the time as seen in Table 3. The probability of crossing a boundary anticipating efficacy is 3.1%, 3.7%, and 2.0% in the Brain, Torso, and Both group. The trial may suggest stopping enrollment for efficacy, but the formal and final efficacy look does not occur until all individuals in that injury group have reached the expected time frame for an observed outcome (i.e., 6 months after randomization for the Brain and Both groups, 1 month after randomization for the Torso group) as described in Section 3.5 above. In the null setting, we force a stop enrolling in the Both arm 11.8% of the time due to ending enrollment in both the Brain and Torso injury groups.

In our hypothesized scenario (Scenario 2), we stop for anticipated efficacy 95.4%, 87.8%, and 31.4% of the time for the Brain, Torso, and Both injury groups, respectively. After accounting for the re-analysis of the Both injury group if Brain and Torso are significant *and* waiting for the full data to be observed, this corresponds to power levels of 94.9%, 82.9%, and 64.4% in the Brain, Torso, and Both injury groups, respectively, as seen in Table 3.

In the mixed scenarios (Scenarios 5-8) and the harmful Brain scenario (Scenario 9), we appropriately stop for futility in null injury groups while minimally stopping for futility in efficacious injury groups.

Table 5: Stopping reasons for individual injuries

| **Scenario** | **Brain** | | | | **Torso** | | | | **Both** | | | | |
| --- | --- | --- | --- | --- | --- | --- | --- | --- | --- | --- | --- | --- | --- |
|  | **Reached N=2000** | **Anticipated Efficacy** | **Futility** | **Max Injury N** | **Reached N=2000** | **Anticipated Efficacy** | **Futility** | **Max Injury N** | **Reached N=2000** | **Anticipated Efficacy** | **Futility** | **Forced Close** | **Max Injury N** |
| 1 | 68.9% | 3.1% | 28.0% | 0.0% | 60.4% | 3.7% | 30.1% | 5.8% | 61.7% | 2.0% | 24.5% | 11.8% | 0.0% |
| 2 | 4.6% | 95.4% | 0.0% | 0.0% | 8.4% | 87.8% | 0.2% | 3.6% | 7.6% | 31.4% | 0.4% | 60.6% | 0.0% |
| 3 | 7.4% | 92.5% | 0.1% | 0.0% | 17.3% | 70.7% | 0.5% | 11.5% | 15.2% | 29.9% | 0.7% | 54.2% | 0.0% |
| 4 | 2.0% | 98.0% | 0.0% | 0.0% | 3.6% | 94.1% | 0.1% | 2.2% | 3.4% | 33.8% | 0.3% | 62.5% | 0.0% |
| 5 | 7.6% | 92.3% | 0.1% | 0.0% | 28.1% | 8.2% | 21.2% | 42.6% | 22.1% | 40.1% | 1.1% | 36.7% | 0.0% |
| 6 | 73.1% | 6.0% | 20.9% | 0.0% | 29.8% | 68.8% | 0.4% | 1.0% | 52.9% | 30.6% | 1.6% | 14.9% | 0.0% |
| 7 | 72.2% | 4.8% | 23.0% | 0.0% | 34.2% | 63.6% | 0.5% | 1.6% | 61.5% | 3.8% | 18.0% | 16.7% | 0.0% |
| 8 | 9.5% | 90.5% | 0.1% | 0.0% | 31.3% | 6.6% | 24.5% | 37.6% | 27.0% | 5.6% | 18.0% | 49.4% | 0.0% |
| 9 | 11.6% | 0.1% | 88.3% | 0.0% | 15.1% | 77.2% | 0.4% | 7.2% | 17.5% | 24.0% | 1.8% | 56.7% | 0.0% |

The RAR allows for doses to temporarily be dropped if their proposed allocation drops below 10%. For each injury dose, the proportion of trials where a dose is temporarily dropped and then restarted within the same simulation is summarized in Table 6. The only way a placebo dose can be dropped within an injury group is if efficacy or futility thresholds are met for that injury group. There is no set time frame that a dose can be temporarily dropped for (e.g., it can be dropped and start again the following interim or it can be dropped and start again several interims later). The 45 mg/kg arm is the dose that is most frequently started and then temporarily stopped. For example, in the null scenario (Scenario 1), the 45 mg/kg arm starts, is temporarily dropped in at least one interim, and then starts again in 15% of the trials. This is likely due to the uncertainty around modeling the 45 mg/kg dose initially in combination with the RAR identifying the ED80.

Table 6: Proportion of trials that temporarily stop then restart a dose

| **Scenario** | **Brain** | | | | **Torso** | | | | **Both** | | | |
| --- | --- | --- | --- | --- | --- | --- | --- | --- | --- | --- | --- | --- |
|  | **Placebo** | **15 mg/kg** | **30 mg/kg** | **45 mg/kg** | **Placebo** | **15 mg/kg** | **30 mg/kg** | **45 mg/kg** | **Placebo** | **15 mg/kg** | **30 mg/kg** | **45 mg/kg** |
| 1 | 0% | 29% | 15% | 15% | 0% | 21% | 17% | 16% | 0% | 16% | 15% | 19% |
| 2 | 0% | 8% | 1% | 38% | 0% | 15% | 2% | 46% | 0% | 19% | 2% | 48% |
| 3 | 0% | 10% | 1% | 37% | 0% | 22% | 4% | 45% | 0% | 22% | 3% | 51% |
| 4 | 0% | 6% | 0% | 37% | 0% | 11% | 2% | 45% | 0% | 15% | 2% | 48% |
| 5 | 0% | 10% | 1% | 39% | 0% | 32% | 12% | 25% | 0% | 26% | 3% | 53% |
| 6 | 0% | 38% | 12% | 20% | 0% | 19% | 4% | 48% | 0% | 26% | 4% | 52% |
| 7 | 0% | 36% | 14% | 18% | 0% | 21% | 5% | 46% | 0% | 22% | 11% | 27% |
| 8 | 0% | 11% | 1% | 39% | 0% | 31% | 14% | 23% | 0% | 25% | 11% | 29% |
| 9 | 0% | 12% | 17% | 3% | 0% | 18% | 6% | 43% | 0% | 19% | 7% | 37% |

## Expected Sample Sizes by Injury Group and Dose

Due to the RAR, the proportions of patients in each study dosing arm change based on the accumulating data. For each simulated trial, we record the sample size per dose at the time that an injury group stops accrual. The median sample sizes for each study arm within each injury group are displayed for the hypothesized scenario (Scenario 2) in Figure 5. The bars extending from each median display the 10^th^ and 90^th^ percentile sample sizes among the simulations. Since RAR does not begin until 500 patients have been randomized, there is a minimum number of patients that are expected to be observed for the placebo, 15 mg/kg, and 30 mg/kg dosing arms in the three injury groups. In this scenario, the assumed effect sizes are 3.6 – 15 mg/kg, 4.5 – 30 mg/kg, 4.9 – 45 mg/kg, so that the ED80 would be the minimum dose that results in an effect size of at least 3.9 QoL units in AUC (4.9 QoL in 45 mg/kg * 80% = 3.9 QoL threshold). In this case, the 30 mg/kg dose would be the correct ED80 in each injury group. As seen in the figure, RAR favors the 30 mg/kg arm as expected.


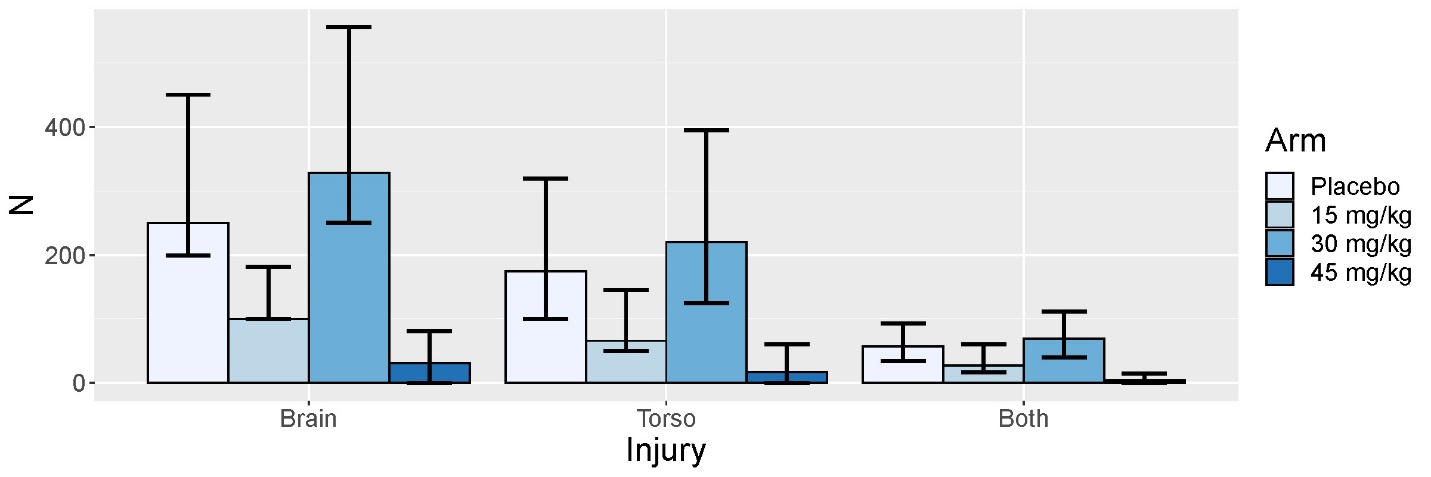


Figure 5: Summaries of expected sample sizes by injury group and arm for the hypothesized scenario (Scenario 2). The bars display the median and the lines display the 10^th^ and 90^th^ percentile of the expected sample sizes.

Figure 6 displays the average sample sizes under the global null scenario (Scenario 1). The adaptive randomization tends to apply roughly equal allocation across the doses. Even though the 45 mg/kg dosing arm is opened in approximately in 20% of the null simulations, very few patients are actually allocated to the 45 mg/kg dose. This is partly due to the restrictions that are placed on the allocation probabilities to the high dose once it is introduced.


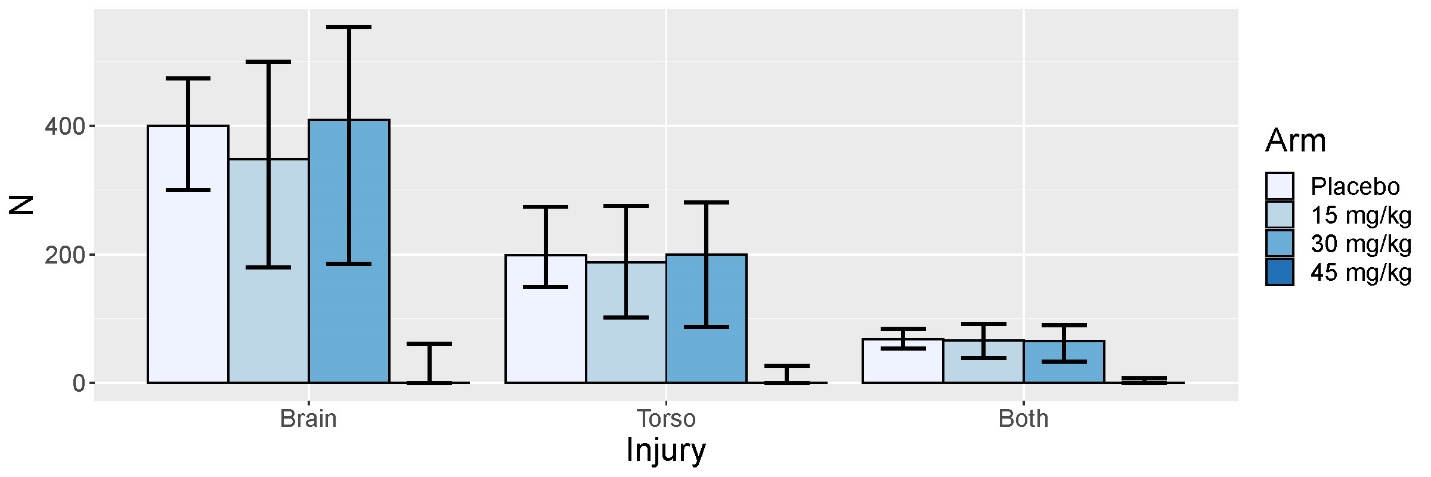


Figure 6: Summaries of expected sample sizes by injury group and arm for the null scenario (Scenario 1). The bars display the median and the lines display the 10^th^ and 90^th^ percentile of the expected sample sizes.

All other expected sample sizes by injury group are presented in Figure 7 through Figure 13.


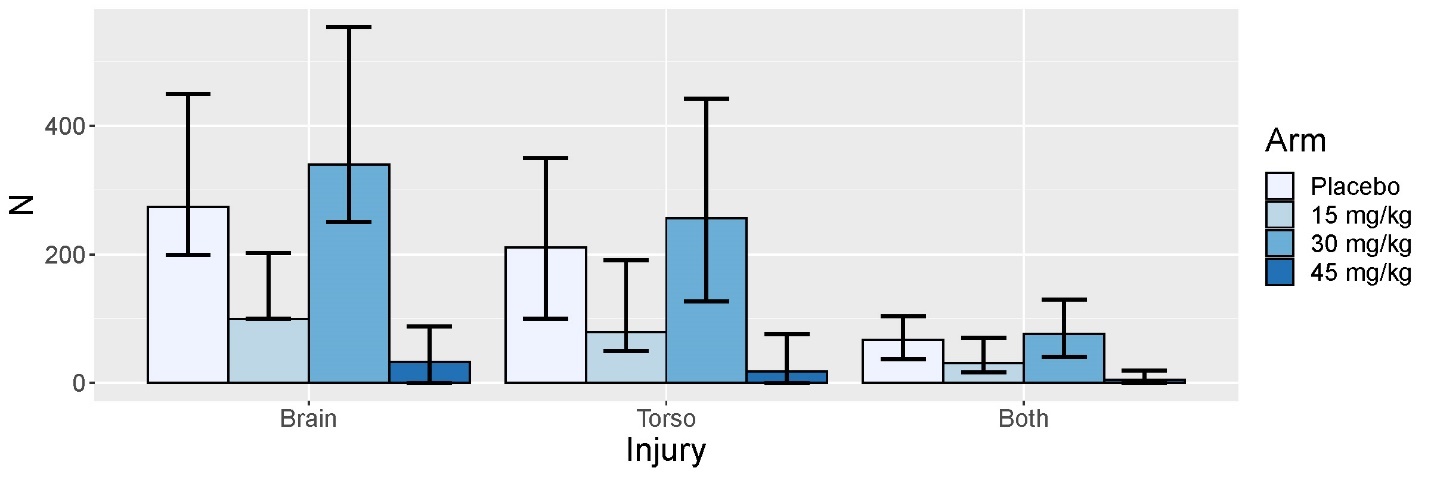


Figure 7: Summaries of expected sample sizes by injury group and arm for Scenario 3


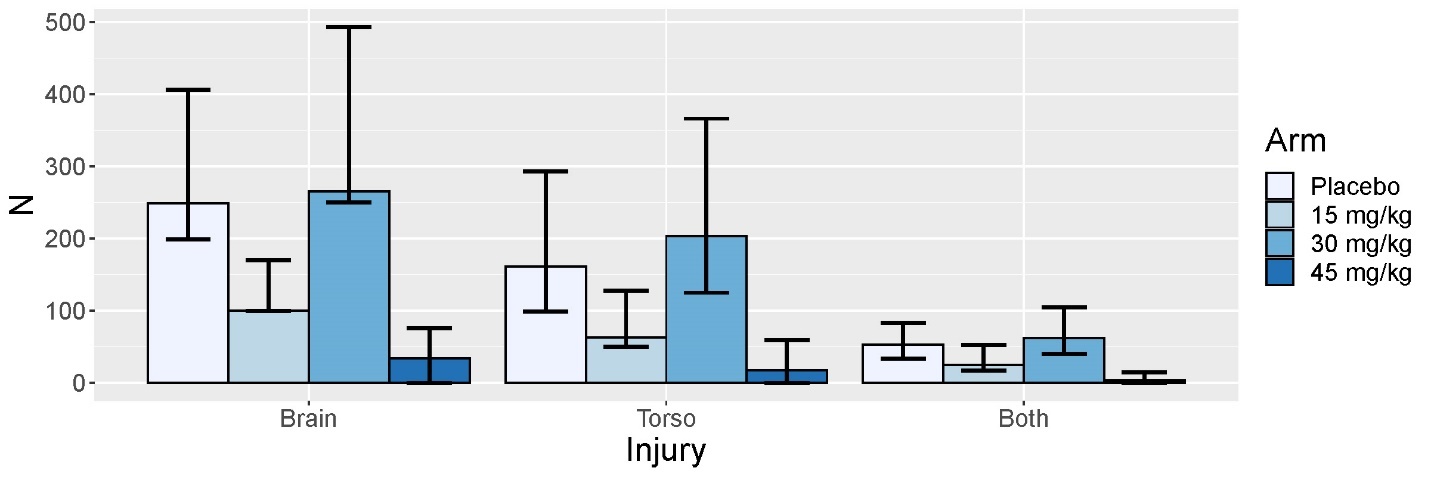


Figure 8: Summaries of expected sample sizes by injury group and arm for Scenario 4

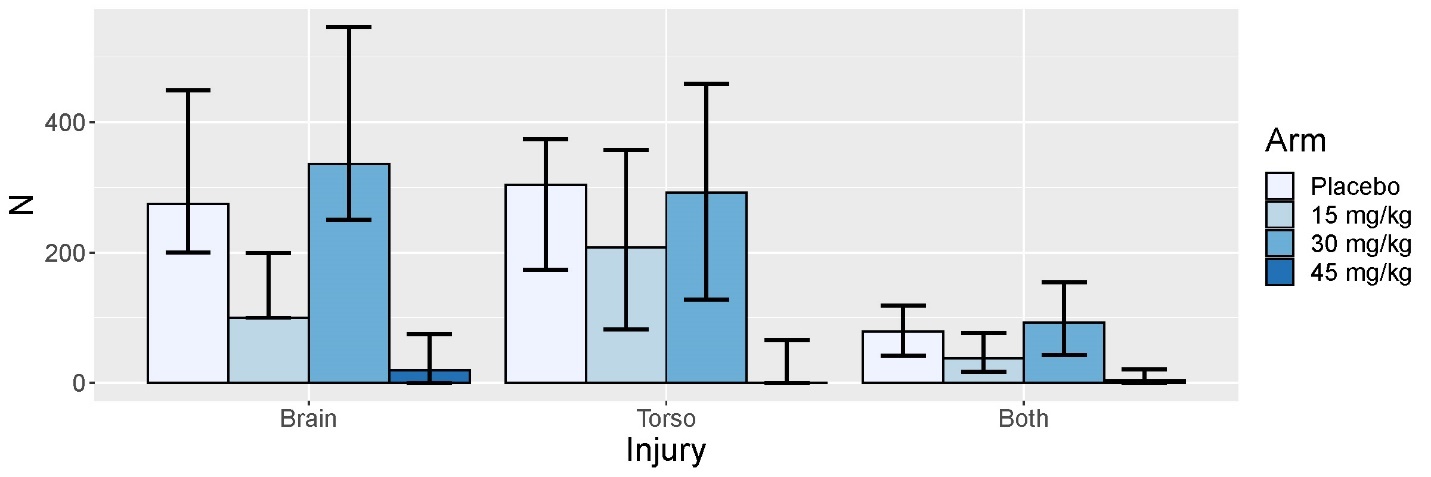


Figure 9: Summaries of expected sample sizes by injury group and arm for Scenario 5


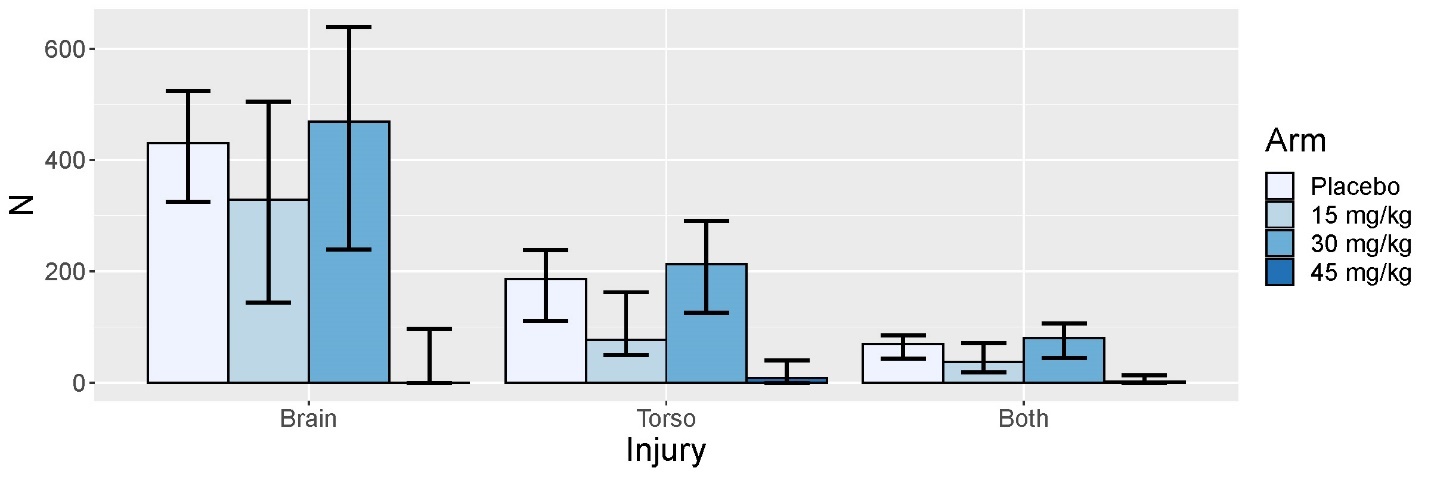


Figure 10: Summaries of expected sample sizes by injury group and arm for Scenario 6


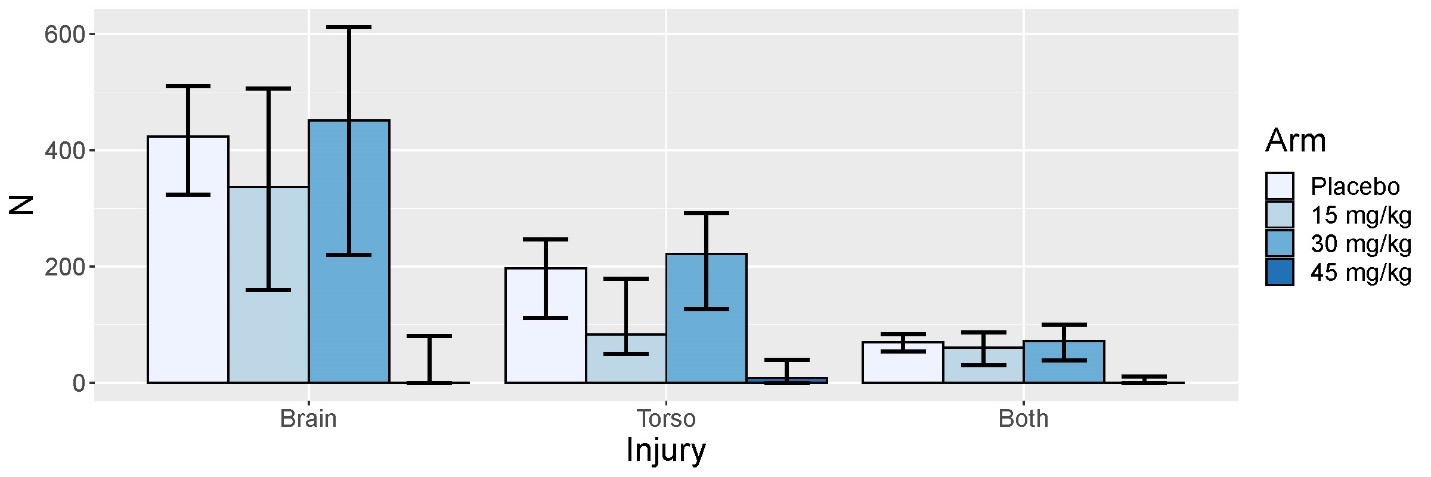


Figure 11: Summaries of expected sample sizes by injury group and arm for Scenario 7


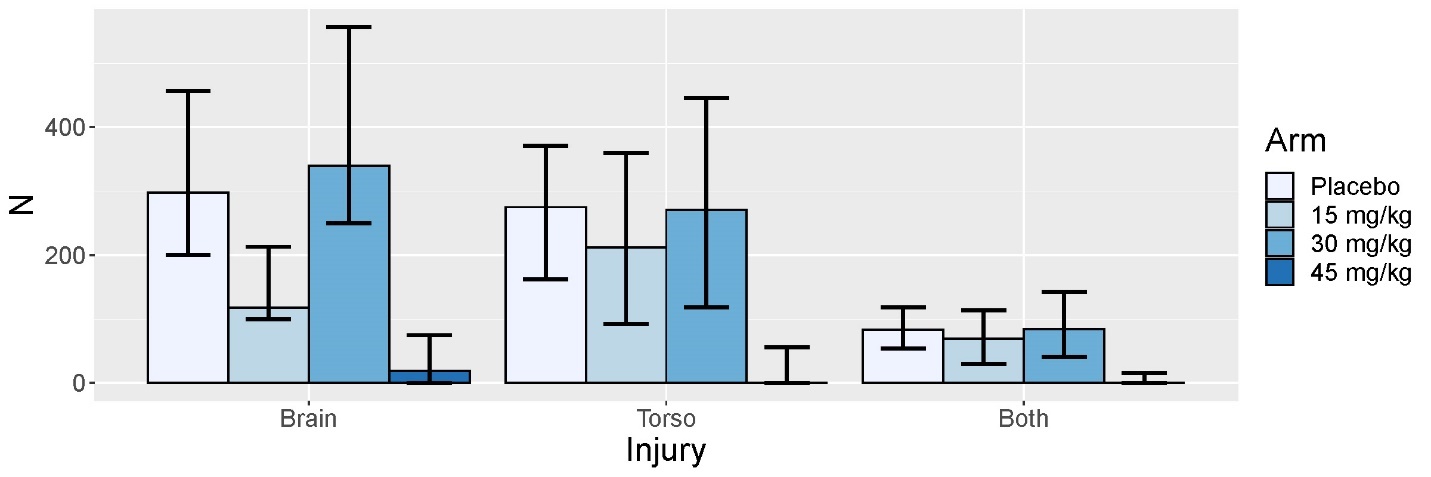


Figure 12: Summaries of expected sample sizes by injury group and arm for Scenario 8


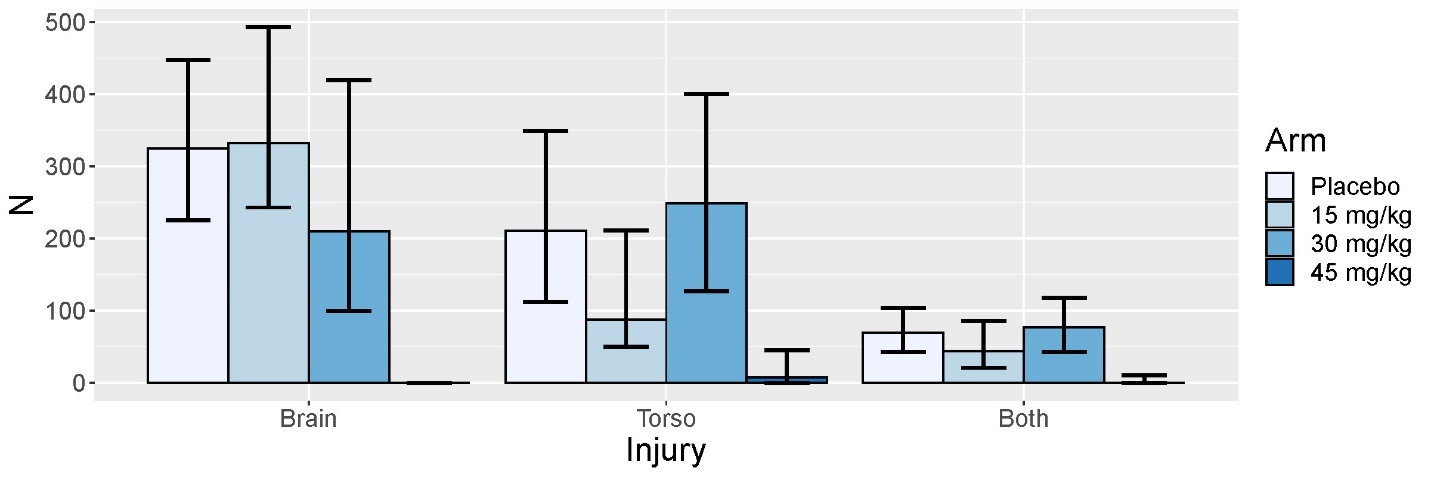


Figure 13: Summaries of expected sample sizes by injury group and arm for Scenario 9

# Limitations of Simulations

There are limitations of the simulations. In the trial, if a patient dies within the follow-up window, his or her AUC will be set to 0 to indicate the worse possible scenario. Due to the complexity of the study design with the borrowing across injury groups, this additional feature (simulating a proportion of deaths and setting the AUC to 0) was not added to the simulations. Another limitation is we assumed a 100% response rate during the simulations. Since the outcome incorporates multiple time points and only one needs to be observed to calculate the AUC, the amount of missingness will be less comparable to a traditional trial (e.g., an outcome at a fixed time point). In the pilot study of 31 patients, 100% of the AUC outcomes were observed. In order to open the 45 mg/kg dose in an injury group, there needs to be potential for a large effect size and there must be no safety concerns from the Data and Safety Monitoring Board. The incorporation of hypothetical adverse events was not included in the simulation to not increase the complexity of the already complex simulations.

There are many methods for analyzing dose response data as discussed in Bretz, Pinheiro, and Branson (2005), but only the hyperbolic $E_{max}$ model was considered. However, a detailed review by Thomas et al. (2014) assessed several dose-response datasets in a pharmaceutical company and they concluded the hyperbolic $E_{max}$ consistently did well at estimating the relationships. The hyperbolic $E_{max}$ has been shown to perform better with multiple doses being considered. In this study, there are only two to three doses of TXA being considered (in addition to the placebo).

# Appendix: Computational Algorithms

The simulations were coded in the R statistical computing environment. The dose-response model was fit using JAGS (Just Another Gibbs Sampler). Posterior quantities are estimated using Markov chain Monte Carlo (MCMC) methods. Posterior samples from the model parameters are then used to estimate additional posterior quantities such as the probability that each dose is the ED80 within an injury group.

For example, in a single iteration of the MCMC, if it is estimated that the 45 mg/kg dose has an increase in AUC compared to placebo of 5 quality of life (QoL) units, the 30 mg/kg dose has an increase of 3.5 QoL units, and the 15 mg/kg dose has an increase of 3.1 QoL units, then the 80% threshold would be 4 QoL units (i.e., $5*0.8$). Among the doses being considered, the smallest dose among the three doses considered that is estimated to have at least 4 QoL units is the $ED_{80}$ for that iteration (in this case the 45 mg/kg dose is the arm chosen). This process is repeated for every MCMC iteration. Any negative estimates for the maximum treatment effect automatically assigns the ‘optimal’ dose to the 15 mg/kg dosing arm to minimize the amount of drug given to patients (i.e., the optimal dose is not assigned to the placebo arm for negative estimates since the placebo arm has a fixed proportion). For each dose, the probability that the dose is the ED80, Pr(d = ED80) is the proportion of MCMC iterations in which that dose was selected.

Berry, S. M., Broglio, K. R., Groshen, S., & Berry, D. A. (2013). Bayesian hierarchical modeling of patient subpopulations: efficient designs of Phase II oncology clinical trials. *Clin Trials, 10*(5), 720-734. doi:10.1177/1740774513497539

Bretz, F., Pinheiro, J. C., & Branson, M. (2005). Combining Multiple Comparisons and Modeling Techniques in Dose-Response Studies. *Biometrics, 61*(3), 738-748. doi:10.1111/j.1541-0420.2005.00344.x

Gajewski, B. J., Meinzer, C., Berry, S. M., Rockswold, G. L., Barsan, W. G., Korley, F. K., & Martin, R. H. (2019). Bayesian hierarchical EMAX model for dose-response in early phase efficacy clinical trials. *Stat Med, 38*(17), 3123-3138. doi:10.1002/sim.8167

Macdougall, J. (2006). Analysis of Dose–Response Studies—Emax Model. In: Ting N. (eds) Dose Finding in Drug Development. New York, NY: Springer.

Thomas, N., Sweeney, K., & Somayaji, V. (2014). Meta-Analysis of Clinical Dose–Response in a Large Drug Development Portfolio. *Statistics in Biopharmaceutical Research, 6*(4), 302-317. doi:10.1080/19466315.2014.924876
